# A Fully Frequency-Dependent Formulation of the Open Quantum System Polarizable Continuum Model

**DOI:** 10.1021/acs.jctc.6c00904

**Published:** 2026-08-12

**Authors:** Gabriel Gil, Stefano Corni, Ciro A. Guido

**Affiliations:** † Dipartimento di Scienze e Innovazione Tecnologica, Università del Piemonte Orientale, Viale T. Michel 11, 15121 Alessandria, Italy; ‡ Instituto de Cibernética, Matemática y Física (ICIMAF), Calle E esquina 15, No. 309, 10400 La Habana, Cuba; § Department of Chemical Sciences, University of Padova, via Marzolo 1, 35100 Padova, Italy; ∥ CNR Institute of Nanoscience, via Campi 213/A, 41100 Modena, Italy

## Abstract

We introduce a fully frequency-dependent formulation of the open quantum system polarizable continuum model (OQS-PCM), as derived from the non-Markovian time-dependent stochastic Schrödinger equation for a polarizable bath in the limit of averaging out the stochastic fluctuations. OQS-PCM­(ω) captures the delayed coupling between solute and solvent electronic dynamics, naturally accounting for polarization, dispersion, and energy dissipation—while bridging the different solvation regimes arising from the distinct solute–solvent electronic time scales. This framework provides a transparent, physically grounded description of van der Waals interactions of a molecule with its environment, as well as the effect of fluctuations on electronic excitations. Simulation of absorption spectra in water and benzene provides insight into the role of environmental time scales in solvatochromism and energy redistribution. These results demonstrate the potential of OQS-PCM­(ω) for predictive modeling of optoelectronic properties, photophysics, and the design of molecular systems strongly influenced by environmental electronic dynamics.

## Graphical abstract

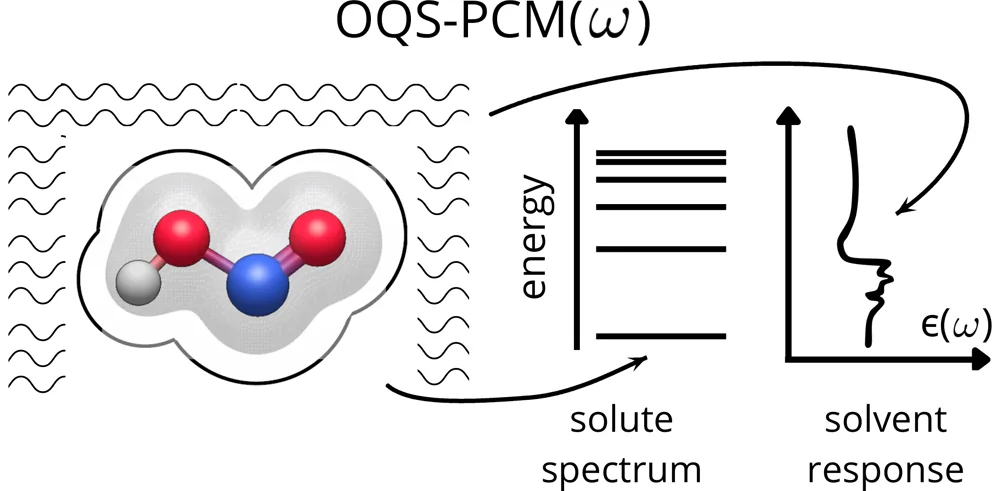

## 1 Introduction

Molecular systems embedded in condensed-phase environments or surrounded by complex media (such as solvents, biological scaffolds, films, aggregates, or inorganic nanoparticles) give rise to most photophysical and photochemical processes of technological and biological relevance. Indeed, the coupling between the target molecule and its environment modifies the electronic structure and energy landscape, affecting spectral features, reactivity and ultrafast dynamics.

Realistic theoretical modeling of such systems remains challenging due to the large differences in the length scales of the target molecule and its surrounding environment, as well as the need to faithfully represent the response of the environment beyond a static screening. Multiscale approaches, where the molecules of interest are treated quantum-mechanically while the environment is modeled either as a continuum dielectric or as an atomistic classical embedding, are effective and promising strategies for addressing these challenges. Despite their widespread use, conventional methodologies of this type have well-known limitations: they assume simplified time-scale separations of the electronic degrees of freedom of the focus molecule and environment, treat solute–solvent weak interactions heuristically, and neglect explicit electronic energy dissipation into the environment due to the coupled electronic dynamics.

Among the continuum approaches, the polarizable continuum model (PCM) has become the most widely adopted method for modeling molecules in isotropic solvent or in anisotropic liquid crystal phase^1^ and it has also been extended to describe molecules in the presence of plasmonic nanoparticles (PCM-NP).^2^ In the PCM framework, the solvent is treated as an infinite dielectric material described by its dielectric constant (for isotropic media) or tensor (if anisotropic environments are described) and the response of the solvent to the molecular electrostatic potential is expressed in terms of polarization charges localized on discrete elements of a molecular-shaped cavity, built from atom-centered interlocking spheres. The model has been also extended to a real-time formulation using a frequency-dependent dielectric function to study the evolution of the molecular density subject to time-dependent perturbations, together with the polarization of the external medium.^3–6^ Extensions of PCM incorporating ad hoc corrections for solute–solvent interactions beyond electrostatics have also been proposed for systems in their electronic ground^7,8^ and excited states.^9–12^ However, they are not on the same theoretical footing as its built-in polarization response.

The accurate theoretical description of solvation effects on electronically excited states remains a nontrivial problem, due to the treatment of nonequilibrium solute–solvent interactions following electronic excitation: indeed, in standard continuum approaches, the nonequilibrium formulation relies on a separation between fast (electronic) and slow (nuclear) solvent polarization components, with the former governed by the optical dielectric constant (ε_∞_). However, from a solute–solvent coupled electronic dynamics perspective, the polarizable models addresses two limits: the self-consistent field and the Born–Oppenheimer^13^ solvation regimes. The first assumes that the electronic degree of freedom of the environment are slower than the solute ones and therefore they respond to the solute mean field: this is how all the polarizable approaches are usually implemented, even in their real-time formulation. Conversely, in the Born–Oppenheimer (recently called “antiadiabatic”^14^) solvation regime, the fast solvent electrons respond instantaneously to the molecular electronic degrees of freedom—an approach that has been used to describe electron-transfer reactions in solution.^13^ However, realistic situations fall usually in between these two opposite solvation regimes. Indeed, even though solvents are usually optically inactive in the visible range, their excitations may overlap with the solute’s near-UV transitions, becoming relevant not only in ultrafast but even in steady-state spectroscopies of solvated systems. In fact, recent experimental studies and compilations of solvent dielectric function profiles over a wide spectral range^15,16^ support such overlap and enable theoretical investigation of its effects.

The PCM framework is structurally similar to the open quantum systems (OQS) approach, where the total Hamiltonian is partitioned into system, bath, and interaction terms, with the latter expressed as a sum of factorized products of system and bath operators.^17^ Due to this similarity, some of the present authors^18^ noted that the QM/PCM framework can be mapped onto an OQS methodology by identifying the molecule as the system, the solvent as the bath, while replacing the classical polarization charges mediating their interaction by their quantum counterparts. In this context, a non-Markovian time-dependent stochastic Schrödinger equation^17^ for a molecule in a polarizable bath (TD-SSE-pol) was derived.^18^ TD-SSE-pol yields an effective, history-dependent electronic evolution of the solvated system, subject to random fluctuations in the solvent degrees of freedom. Writing TD-SSE-pol in the frequency domain and averaging the solvent fluctuations leads to the closely related stationary formulation we called OQS-PCM. Reference 18 laid the foundations of both TD-SSE-pol and OQS-PCM, but focused on an approximate frequency-dependent response of the bath and weakly polar solvents, thereby constraining the range of accessible applications.

In this contribution, we present a state-specific implementation of the OQS-PCM approach, OQS-PCM­(ω), that *fully* incorporates the frequency-dependent response of generic-polarity solvents, along with polarization, dispersion, and dissipation effects. Our methodology accounts for these effects not only in the ground and excited states energies, but also in transition properties such as the oscillator strengths.

To illustrate the advantages of the OQS-PCM­(ω) implementation, we computed the absorption spectra of small molecules (hydrochloric acid, formaldehyde, nitrous acid) in solvents of very different polarity (water and benzene). In these examples, the method captures pronounced solvent-dependent and state-specific spectral shifts that are overlooked by the standard PCM implementations. Notably, OQS-PCM­(ω) results do not reduce to a trivial interpolation between the self-consistent and Born–Oppenheimer regimes, underscoring the need for a general treatment of solute–solvent time scales. Importantly, we found that the Born–Oppenheimer (or *antiadiabatic*) solvation approach significantly overestimates dispersion effects in both ground and excited states.

The paper is structured as follows. First, we summarize the OQS-PCM theory with an emphasis on the generic polarity treatment, which can be readily applied to both weakly and strongly polar solvents.^18^ This section also introduces the limiting regimes of OQS-PCM and the approximate approaches considered throughout the paper. Next, we present the full-frequency implementation of OQS-PCM, highlighting its state-specific character. We then briefly summarize the computational methodology. The Results section illustrates the method through applications to nitrous acid in water and benzene, as well as formaldehyde and hydrochloric acid in water. It analyzes polarization, dispersion, and dissipation effects (Section 5.1), explores the solute–solvent limiting time scales (Section 5.2) and the performance of the approximate and full-frequency OQS-PCM­(ω) approaches (Section 5.3), examines the influence of solvent polarity on solvatochromic shifts (Section 5.4), and compares our results with experimental data for the test molecules (Section 5.5). In addition, a convergence study of the ground-state solvated energy provides insight into why the Born–Oppenheimer approximation departs significantly from the full OQS-PCM­(ω) treatment (Section 5.6). A detailed account of our computational protocols is provided in Section S2 of the Supporting Information, which also contains an extended theoretical discussion (Section S1) and additional data for our prototypical systems (Section S3). Finally, the *Concluding Remarks* Section 6 summarizes the main methodological developments and physical insights arising from the present work.

## 2 The Open Quantum System Polarizable Continuum Model

Within the open quantum systems (OQS) theory framework, the *full system* (e.g., a solvated molecule) is divided into a *focus subsystem* (e.g., a solute) and a *bath* (e.g., a solvent). In contrast to the standard polarizable continuum model (PCM), in which the bath is treated as a classical medium from the outset, in the OQS approach, the bath degrees of freedom are considered within quantum mechanics, and then properly traced out by leveraging the quantum statistical typicality assumption.^17^ Here, we will explicitly focus on the bath’s electronic degrees of freedom.

We may write the full Hamiltonian of the open quantum system as

1 $$Ĥ={Ĥ}_{S}⊗{Î}_{B}+{Î}_{S}⊗{Ĥ}_{B}+{Ĥ}_{SB}$$

where ${Ĥ}_{S}$ and ${Ĥ}_{B}$ are the Hamiltonians of the isolated focus subsystem and bath, whereas ${Ĥ}_{SB}$ is the interaction Hamiltonian between them. ${Î}_{B}$ and ${Î}_{S}$ are ancillary identity operators acting on either the bath or the subsystem state subspaces. If the above factorization is physically meaningful (as is typically the case for molecules in solution), we may write the interaction Hamiltonian as

2 $${Ĥ}_{SB}=\sum_{\alpha }{Ŝ}_{\alpha }⊗{\mathrm{B̂}}_{\alpha }$$

where ${Ŝ}_{\alpha }$ is an operator acting on the subsystem state, whereas ${\mathrm{B̂}}_{\alpha }$ is the corresponding operator acting on the bath state. Hereafter, we refer to the focus subsystem as the system *simpliciter*.

Following Gaspard and Nagaoka,^17^ it is possible to obtain a non-Markovian stochastic Schrödinger equation for the system state, including a random and a history-dependent term, from the Hamiltonian in eqs 1 and 2. In atomic units, it reads

3 $$\begin{array}{l} i\;\frac{\partial }{\partial t}|\Psi \left(t\right)\rangle ={Ĥ}_{S}|\Psi \left(t\right)\rangle +\sum_{\alpha }\eta_{\alpha }\left(t\right){Ŝ}_{\alpha }|\Psi \left(t\right)\rangle +\\ -i\int_{-\infty }^{t}dt^{\prime }\sum_{\alpha ,\beta }\left\langle {\mathrm{B̂}}_{\alpha }\left(t\right){\mathrm{B̂}}_{\beta }\left(t^{\prime }\right)\right\rangle {Ŝ}_{\alpha }\;e^{-i{Ĥ}_{S}\left(t-t^{\prime }\right)}{Ŝ}_{\beta }|\Psi \left(t^{\prime }\right)\rangle \end{array}$$

where η_α_(*t*) denotes a time-dependent random variable with zero expectation value at every time, bath operators are written in the interaction picture, and ⟨···⟩ indicates an expectation value over a *typical* state of the bath. Furthermore, according to the quantum statistical typicality assumption, a quantum-mechanical expectation value over a typical bath state may be approximated by the corresponding thermal average. As a consequence

4 $$\left\langle {\mathrm{B̂}}_{\alpha }\left(t\right){\mathrm{B̂}}_{\beta }\left(t^{\prime }\right)\right\rangle \approx C_{\alpha \beta }\left(t-t^{\prime }\right)$$

where *C*_αβ_(*t* – *t*′) is the time-dependent bath correlation function (see discussion in ref 17 and references therein). Substituting eq 4 in [Disp-formula eq3], we finally obtain

5 $$\begin{array}{l} i\;\frac{\partial }{\partial t}|\Psi \left(t\right)\rangle ={Ĥ}_{S}|\Psi \left(t\right)\rangle +\sum_{\alpha }\eta_{\alpha }\left(t\right){Ŝ}_{\alpha }|\Psi \left(t\right)\rangle +\\ -i\;\int_{-\infty }^{t}dt^{\prime }\sum_{\alpha ,\beta }C_{\alpha \beta }\left(t-t^{\prime }\right){Ŝ}_{\alpha }\;e^{-i{Ĥ}_{S}\left(t-t^{\prime }\right)}{Ŝ}_{\beta }|\Psi \left(t^{\prime }\right)\rangle \end{array}$$

Importantly, the derivation leading to eqs 3–5 assumes $\left\langle {\mathrm{B̂}}_{\alpha }\right\rangle =0$ .^17^

As noted by Guido et al.,^18^ eq 5 can be suitably mapped onto the PCM framework as follows. The system operator ${Ŝ}_{\alpha }$ corresponds to the solute potential evaluated at the *i*-th tessera of the solvent-excluded cavity surface, i.e., ${\mathrm{V̂}}_{i}\equiv \mathrm{V̂}\left(r_{i}\right)$ ; and the bath operator ${\mathrm{B̂}}_{\alpha }$ mirrors the quantum counterpart of the solvent polarization charge at that same tessera, i.e., ${\mathrm{q̂}}_{i}$ .

For a solvated system in a solvent of generic polarity $\left\langle {\mathrm{q̂}}_{i}\right\rangle \neq 0$ , and the expressions analogous to ${Ĥ}_{S}$ and ${Ĥ}_{SB}$ can be recast as follows^17,18^

6 $${\hat{\overset{®}{H}}}_{S}={Ĥ}_{S}+\sum_{i}{\mathrm{V̂}}_{i}\left\langle {\mathrm{q̂}}_{i}\right\rangle$$

7 $${\hat{\overset{®}{H}}}_{SB}=\sum_{i}{\mathrm{V̂}}_{i}⊗\left({\mathrm{q̂}}_{i}-\left\langle {\mathrm{q̂}}_{i}\right\rangle \right)$$

where the original bath operator ${\mathrm{B̂}}_{\alpha }$ is mapped onto ${\hat{\overset{®}{q}}}_{i}={\mathrm{q̂}}_{i}-\left\langle {\mathrm{q̂}}_{i}\right\rangle$ to guarantee it averages to zero. Average polarization charges $\left\langle {\mathrm{q̂}}_{i}\right\rangle$ are assumed to correspond to inertial charges (as discussed in the Supporting Information Section S1.2), since here we explicitly focus on the bath’s electronic degrees of freedom. Note that both ${\mathrm{q̂}}_{i}$ (and ${\hat{\overset{®}{q}}}_{i}$ ) act on the Hilbert space of solvent states. Additionally, we may write the solvent correlation function as

8 $$\left\langle {\hat{\overset{®}{q}}}_{i}\left(t\right){\hat{\overset{®}{q}}}_{j}\left(t^{\prime }\right)\right\rangle \approx C_{ij}\left(t-t^{\prime }\right)=C_{ij}^{sym}\left(t-t^{\prime }\right)+iQ_{ij}\left(t-t^{\prime }\right)/2$$

where *Q*_*ij*_(*t* – *t*′) is the time-dependent PCM response matrix, and *C*_*ij*_^sym^(*t* – *t*′), the symmetric correlation function of the environment.^18,19^ Therefore, mapping eq 5 into the PCM framework produces a stochastic Schrödinger equation (SSE) that includes dissipation via *C*_*ij*_^sym^(*t* – *t*′) and the imaginary part of *Q*_*ij*_(*t* – *t*′), i.e.

9 $$\begin{array}{l} i\;\frac{\partial }{\partial t}|\Psi \left(t\right)\rangle ={\hat{\overset{®}{H}}}_{S}|\Psi \left(t\right)\rangle +\sum_{i}\eta_{i}\left(t\right){\mathrm{V̂}}_{i}|\Psi \left(t\right)\rangle +\\ -i\int_{-\infty }^{t}dt^{\prime }\sum_{i,j}C_{ij}^{sym}\left(t-t^{\prime }\right){\mathrm{V̂}}_{i}\;e^{-i{\hat{\overset{®}{H}}}_{S}\left(t-t^{\prime }\right)}{\mathrm{V̂}}_{j}|\Psi \left(t^{\prime }\right)\rangle +\\ +\frac{1}{2}\int_{-\infty }^{t}dt^{\prime }\sum_{i,j}Q_{ij}\left(t-t^{\prime }\right){\mathrm{V̂}}_{i}\;e^{-i{\hat{\overset{®}{H}}}_{S}\left(t-t^{\prime }\right)}{\mathrm{V̂}}_{j}|\Psi \left(t^{\prime }\right)\rangle \end{array}$$

Equation 9 constitutes the theoretical core of the time-dependent SSE for a polarizable continuum bath (TD-SSE-pol). As in ref 18, we neglect its stochastic contribution in moving to the stationary formulation—an approximation justified below and further detailed in Section S1.1 of the Supporting Information.

Introducing the resolution of identity in terms of the eigenstates of the *modified* system Hamiltonian of eq 6, and applying the definition of the Fourier transform for the PCM response matrix, as well as the fluctuation–dissipation theorem,^19^ we get the following time-independent Schrödinger equation for stationary states^18^

10 $$\begin{array}{l} {\hat{\overset{®}{H}}}_{S}|\Phi_{A}^{\prime }\rangle +\frac{1}{2}\sum_{K}\sum_{ij}R\left\{Q_{ij}\left({\mathrm{\omega ̅}}_{A^{\prime }K}\right)\right\}{\mathrm{V̂}}_{i}|{\mathrm{\Phi ̅}}_{K}\rangle \left\langle {\mathrm{\Phi ̅}}_{K}\left|{\mathrm{V̂}}_{j}\right|\Phi_{A}^{\prime }\right\rangle \\ +\frac{i}{2}\sum_{K}T\left({\mathrm{\omega ̅}}_{A^{\prime }K}\right)\sum_{ij}I\left\{Q_{ij}\left({\mathrm{\omega ̅}}_{A^{\prime }K}\right)\right\}{\mathrm{V̂}}_{i}|{\mathrm{\Phi ̅}}_{K}\rangle \left\langle {\mathrm{\Phi ̅}}_{K}\left|{\mathrm{V̂}}_{j}\right|\Phi_{A}^{\prime }\right\rangle =E_{A}^{\prime }|\Phi_{A}^{\prime }\rangle \end{array}$$

In this equation, |Φ_*A*_^′^⟩ and *E*_*A*_^′^ are the solvated OQS-PCM stationary state and energy, whereas $|{\mathrm{\Phi ̅}}_{K}\rangle$ and ${\mathrm{E̅}}_{K}$ are the eigenstates and eigenenergies of ${\hat{\overset{®}{H}}}_{S}$ (i.e., the molecular Hamiltonian in equilibrium with the solvent’s inertial charges), and ${\mathrm{\omega ̅}}_{A^{\prime }K}=E_{A}^{\prime }-{\mathrm{E̅}}_{K}$ . $R\left\{Q_{ij}\left(\omega \right)\right\}$ and $I\left\{Q_{ij}\left(\omega \right)\right\}$ are the real and imaginary parts of the frequency-dependent PCM response matrix, respectively. Furthermore, in the low temperature limit, *T*(ω) ≈ 1 + Θ­(ω) – Θ­(−ω), with Θ­(ω) denoting the Heaviside step function. The state-specific Hamiltonian operator for a state *A*′ is therefore

11 $$\begin{array}{l} {Ĥ}_{OQS}^{A^{\prime }}={\hat{\overset{®}{H}}}_{S}+\frac{1}{2}\sum_{K}\sum_{ij}R\left\{Q_{ij}\left({\mathrm{\omega ̅}}_{A^{\prime }K}\right)\right\}{\mathrm{V̂}}_{i}|{\mathrm{\Phi ̅}}_{K}\rangle \langle {\mathrm{\Phi ̅}}_{K}|{\mathrm{V̂}}_{j}\\ +\frac{i}{2}\sum_{K}T\left({\mathrm{\omega ̅}}_{A^{\prime }K}\right)\sum_{ij}I\left\{Q_{ij}\left({\mathrm{\omega ̅}}_{A^{\prime }K}\right)\right\}{\mathrm{V̂}}_{i}|{\mathrm{\Phi ̅}}_{K}\rangle \langle {\mathrm{\Phi ̅}}_{K}|{\mathrm{V̂}}_{j} \end{array}$$

Expanding the solvated states in terms of $|{\mathrm{\Phi ̅}}_{J}\rangle$ , i.e., $|\Phi_{A}^{\prime }\rangle =\sum_{J}C_{J}^{A^{\prime }}|{\mathrm{\Phi ̅}}_{J}\rangle$ , and projecting on a particular state $\langle {\mathrm{\Phi ̅}}_{I}|$ , we arrive at^18^

12 $$\sum_{J}H_{OQS,IJ}^{A^{\prime }}C_{J}^{A^{\prime }}=E_{A}^{\prime }C_{I}^{A^{\prime }}$$

where

13 $$\begin{array}{l} H_{OQS,IJ}^{A^{\prime }}={\mathrm{E̅}}_{I}\delta_{IJ}+\frac{1}{2}\sum_{K}\sum_{i,j}R\left\{Q_{ij}\left({\mathrm{\omega ̅}}_{A^{\prime }K}\right)\right\}\left\langle {\mathrm{\Phi ̅}}_{I}\left|{\mathrm{V̂}}_{i}\right|{\mathrm{\Phi ̅}}_{K}\right\rangle \left\langle {\mathrm{\Phi ̅}}_{K}\left|{\mathrm{V̂}}_{j}\right|{\mathrm{\Phi ̅}}_{J}\right\rangle \\ +\frac{i}{2}\sum_{K}T\left({\mathrm{\omega ̅}}_{A^{\prime }K}\right)\sum_{i,j}I\left\{Q_{ij}\left({\mathrm{\omega ̅}}_{A^{\prime }K}\right)\right\}\left\langle {\mathrm{\Phi ̅}}_{I}\left|{\mathrm{V̂}}_{i}\right|{\mathrm{\Phi ̅}}_{K}\right\rangle \left\langle {\mathrm{\Phi ̅}}_{K}\left|{\mathrm{V̂}}_{j}\right|{\mathrm{\Phi ̅}}_{J}\right\rangle \end{array}$$

are the matrix elements of the OQS-PCM Hamiltonian eq 11 in the basis of the eigenstates of ${\hat{\overset{®}{H}}}_{S}$ . The first term in the solute–solvent interaction Hamiltonian includes both polarization and dispersion contributions, as disentangled and discussed in ref 18. Recently, an effective methodology rooted in these contributions has been proposed for a polarizable QM/MM approach.^20^ The dispersion effects accounted for in eq 13 arise from the solvent’s response to solute fluctuations, whereas their counterparts—stemming from solute’s response to solvent fluctuations—are described by the stochastic term in the TD-SSEpol,^18^ earlier neglected in the derivation of eq 10. In Section S1.1 of the Supporting Information, we complement this treatment by proving that the stochastic contribution to eq 9 vanishes for a stationary ansatz, after ensemble averaging over environmental fluctuations, thereby establishing eq 10 as an appropriate physical limit of the full SSE. The second interaction term in eq 13 is associated with energy dissipation from the molecule to the environment.

Equation 11 defines a state-specific Hamiltonian, as it depends on the energy of the target solvated state being corrected. Physically, this means that each solute state experiences a distinct response from the solvent. Notice that this state-specificity affects all solute–solvent interactions, including polarization, dispersion, and dissipation (see Figure 1 for an illustration). In practice, this implies that both the energy and the wave function of each state must be determined independently, by diagonalizing eq 13 for that specific state. Moreover, the eigenproblem defined by eqs 12 and 13 is nonlinear, since the Hamiltonian depends on the solvated-state energy obtained from its own diagonalization. As a consequence, the problem must be solved iteratively (see Section 3 and Supporting Information), starting from the eigenvalues of the modified system Hamiltonian.

**Figure 1:**
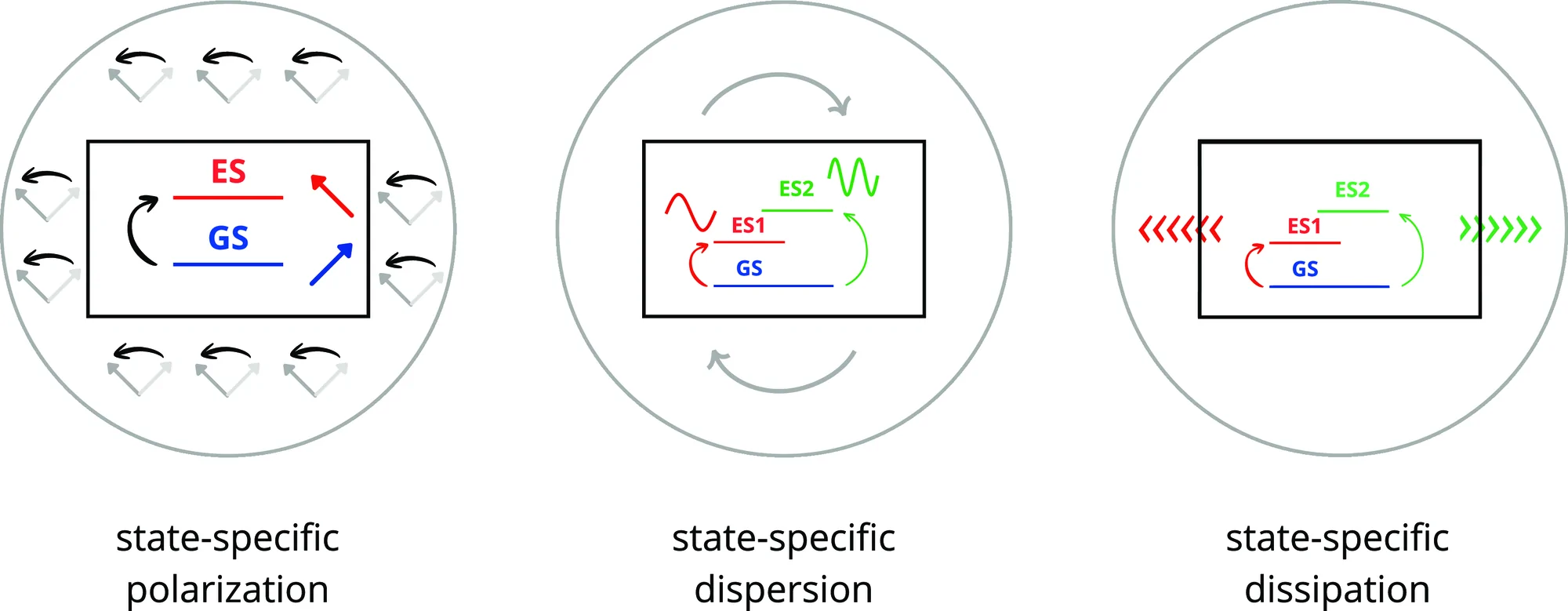
Conceptual illustration of solvation effects in OQS-PCM­(ω). From left to right: state-specific polarization (solvent adapts to each solute state), state-specific dispersion (solvent responds to each solute transition), and state-specific dissipation (each solute transition has a distinct decay channel). The solute is depicted as energy levels within the inner rectangle, and the solvent as a surrounding circle.

Finally, due to the dissipation term, Hamiltonian [Disp-formula eq13] is non-Hermitian, yielding complex eigenvalues. The real part corresponds to the solvation-corrected energy *E*_*A*_^′^, while the imaginary part reflects the finite lifetime of the excited state associated with electronic energy transfer to the solvent. Owing to the real symmetric matrix elements of the electrostatic potentials, Hamiltonian [Disp-formula eq13] is also complex symmetric. Therefore, solving a single eigenproblem for the right eigenvectors, as in eq 12, is sufficient to determine all state-specific and transition properties.

### 2.1 Limiting Regimes and Associated Approximations in OQS-PCM­(ω)

In order to aid in the interpretation and assessment of the results of the general OQS-PCM­(ω) approach, we also introduce here three approximations of the fully frequency-dependent method developed, obtained from a simplified treatment of the frequency dependence of the solvent response. Two correspond to well-known limiting cases of OQS-PCM­(ω), namely the Born–Oppenheimer (*antiadiabatic*) and the self-consistent solvation regimes, which lie at opposite extremes of the solute–solvent time scale,^13,21^ and therefore serve as useful reference points for comparison. The last scheme, hereafter referred to as the *cutoff* approximation, adopts a simplified frequency dependence that qualitatively reproduces the zero- and high-frequency limits of the empirical solvent response. It interpolates between the Born–Oppenheimer and self-consistency regimes. This cutoff approximation depends only on two parameters and can therefore be a useful alternative to OQS-PCM­(ω) when only partial information about the frequency dependence of the solvent dielectric function is available. For ease of presentation, we discuss it first.

#### 2.1.1 Cutoff Approximation

Within the cutoff approximation, a frequency threshold is established above which the solvent cannot respond to solute excitations (fast fluctuations in the solute), and below which the solvent response is uniform.^18^ Additionally, it is assumed that there are no solute–solvent dissipation channels. With such premises, it yields

14 $$Q_{ij}\left(\omega \right)\approx Q_{d,ij}\Theta \left(\omega_{cutoff}-\left|\omega \right|\right)$$

where ω_cutoff_ ≫ 0. Taking the modulus of ω is necessary to preserve the parity of $R\left\{Q_{ij}\left(\omega \right)\right\}$ . *Q*_d,*ij*_ is the (real-valued) dynamic PCM matrix, depending on the dynamic (high-frequency or optical) dielectric constant ϵ_d_ and representing an average solvent response in the optical range. This regime holds approximately when the solvent electronic excitations lie above ω_cutoff_. In such a case, the solvent electrons can follow slower but not faster electronic movements in the solute, associated with low-lying and highly excited states, respectively.

Given the fact that we are already approximating the frequency dependence of the response function, we may assume as well that $Q_{ij}\left({\mathrm{\omega ̅}}_{A^{\prime }K}\right)\approx Q_{ij}\left({\mathrm{E̅}}_{A}-{\mathrm{E̅}}_{K}\right)$ , which is valid whenever $E_{A}^{\prime }\approx {\mathrm{E̅}}_{A}$ , and then apply eq 14 to the latter expression. In this approximation, the matrix elements of OQS-PCM Hamiltonian of [Disp-formula eq13] reduce to

15 $${\mathrm{H̅}}_{OQS,IJ}^{A^{\prime }}={\mathrm{E̅}}_{I}\delta_{IJ}+\frac{1}{2}\sum_{i,j}Q_{d,ij}\sum_{K:\left|{\mathrm{E̅}}_{K}-{\mathrm{E̅}}_{A}\right|\leq \omega_{cutoff}}\left\langle {\mathrm{\Phi ̅}}_{I}\left|{\mathrm{V̂}}_{i}\right|{\mathrm{\Phi ̅}}_{K}\right\rangle \left\langle {\mathrm{\Phi ̅}}_{K}\left|{\mathrm{V̂}}_{j}\right|{\mathrm{\Phi ̅}}_{J}\right\rangle$$

where the sum over *K* is restricted to states from the basis satisfying $\left|{\mathrm{E̅}}_{K}-{\mathrm{E̅}}_{A}\right|\leq \omega_{cutoff}$ . Note that, just as [Disp-formula eq13], the Hamiltonian in [Disp-formula eq15] is state-specific. Thus, we need to diagonalize [Disp-formula eq15] for each solvated state *A*′, and take *the proper* eigenenergy and eigenstate of ${\mathrm{H̅}}_{OQS,IJ}^{A^{\prime }}$ (just one) in order to obtain the solvated spectrum.

#### 2.1.2 Born–Oppenheimer Solvation Regime

We arrive at the Born–Oppenheimer (BO) solvation regime,^13,21^ also recently referred to as the *antiadiabatic* approximation,^14^ by assuming that solvent electrons are strictly faster than those of the solute. In practice, this amounts to take ω_cutoff_ → ∞ in eq 15, i.e.

16 $$Q_{ij}\left(\omega \right)\approx Q_{d,ij}$$

meaning that the solvent is able to respond to any excitation of the solute, and it responds to them in an average way dictated by the dynamical PCM response matrix. The OQS-PCM Hamiltonian in this case results in

17 $${\mathrm{H̅}}_{OQS,IJ}^{A^{\prime }}={\mathrm{E̅}}_{I}\delta_{IJ}+\frac{1}{2}\sum_{i,j}Q_{d,ij}\sum_{K}\left\langle {\mathrm{\Phi ̅}}_{I}\left|{\mathrm{V̂}}_{i}\right|{\mathrm{\Phi ̅}}_{K}\right\rangle \left\langle {\mathrm{\Phi ̅}}_{K}\left|{\mathrm{V̂}}_{j}\right|{\mathrm{\Phi ̅}}_{J}\right\rangle$$

Note that in all three cases, 13, 15 and 17 the summation over *K* is limited to the chosen size of the basis set. Additionally, all of these approaches include both polarization and dispersion effects, as disentangled and identified in ref 18.

The validity of the Born–Oppenheimer solvation regime can be readily understood by comparing eq 17 with its OQS-PCM­(ω) counterpart, eq 13. Since the electronic contribution to the dielectric function at zero frequency is close to its optical-limit value, *Q*_*ij*_(0) ≈ *Q*_d,*ij*_. Therefore, if all the solute electronic energy differences entering the solvent response functions in eq 13 are *sufficiently small* in absolute value, the OQS-PCM­(ω) Hamiltonian approaches the Born–Oppenheimer solvation limit, eq 17. In practice, this requires $\left|E_{A}^{\prime }-{\mathrm{E̅}}_{K}\right|\ll \omega_{cutoff}$ for every *A* and *K*, where ω_cutoff_ denotes a characteristic onset of the solvent electronic response.

#### 2.1.3 Self-Consistent Field Solvation Regime

Finally, when the solvent electrons are much slower than the solute’s one, we recover the self-consistent field solvation regime. Again, starting from eq 15, this amounts to consider ω_cutoff_ ≈ 0, leading to

18 $${\mathrm{H̅}}_{OQS,IJ}^{A^{\prime }}={\mathrm{E̅}}_{I}\delta_{IJ}+\frac{1}{2}\sum_{i,j}Q_{d,ij}\left\langle {\mathrm{\Phi ̅}}_{I}\left|{\mathrm{V̂}}_{i}\right|{\mathrm{\Phi ̅}}_{A}\right\rangle \left\langle {\mathrm{\Phi ̅}}_{A}\left|{\mathrm{V̂}}_{j}\right|{\mathrm{\Phi ̅}}_{J}\right\rangle$$

When we take the diagonal approximation over the latter, we arrive at the following solvated energies

19 $$E_{A}^{\prime }={\mathrm{E̅}}_{A}+\frac{1}{2}\sum_{i,j}Q_{d,ij}\left\langle {\mathrm{\Phi ̅}}_{A}\left|{\mathrm{V̂}}_{i}\right|{\mathrm{\Phi ̅}}_{A}\right\rangle \left\langle {\mathrm{\Phi ̅}}_{A}\left|{\mathrm{V̂}}_{j}\right|{\mathrm{\Phi ̅}}_{A}\right\rangle$$

which is a well-known expression for the free energy including the state-specific polarization in the standard PCM approach. Hence, the Hamiltonian [Disp-formula eq18] only includes polarization effects (no dispersion). However, unlike [Disp-formula eq18], the standard state-specific PCM Hamiltonian producing similar results to 19 is self-consistent through the $\left\langle \Phi_{A}^{\prime }\left|{\mathrm{V̂}}_{i}\right|\Phi_{A}^{\prime }\right\rangle$ -dependency, i.e.

20 $$H_{IJ}^{A^{\prime }}={\mathrm{E̅}}_{I}\delta_{IJ}+\sum_{i,j}Q_{d,ij}\left\langle \Phi_{A}^{\prime }\left|{\mathrm{V̂}}_{i}\right|\Phi_{A}^{\prime }\right\rangle \left\langle {\mathrm{\Phi ̅}}_{I}\left|{\mathrm{V̂}}_{j}\right|{\mathrm{\Phi ̅}}_{J}\right\rangle$$

which means that its diagonalization has to be tackled iteratively. It is also noteworthy that, as a result of coming from a theory of *closed* quantum systems, the energies stemming from the diagonalization of [Disp-formula eq20] are not free energies and have to be corrected by adding the work spent in placing on the cavity the dynamic polarization charges in equilibrium with the state *A*′, i.e.

$$-\frac{1}{2}\sum_{i,j}Q_{d,ij}\left\langle \Phi_{A}^{\prime }\left|{\mathrm{V̂}}_{i}\right|\Phi_{A}^{\prime }\right\rangle \left\langle \Phi_{A}^{\prime }\left|{\mathrm{V̂}}_{j}\right|\Phi_{A}^{\prime }\right\rangle$$

As in the previous section, the validity of the self-consistent field solvation regime can be understood by comparing eq 18 with its OQS-PCM­(ω) counterpart, eq 13. First, note that $E_{A}^{\prime }\approx {\mathrm{E̅}}_{A}$ , since solvation effects can be regarded as perturbatively small. Therefore, $Q_{ij}\left(E_{A}^{\prime }-{\mathrm{E̅}}_{A}\right)\approx Q_{ij}\left(0\right)\approx Q_{d,ij}$ , and the contribution of the *K* = *A* terms in eq 13 reduces to eq 18. Second, at high enough frequencies, the solvent electronic response vanishes, so that *Q*_*ij*_(ω) ≈ 0. Consequently, if the solute electronic energy differences entering the solvent response functions are *sufficiently large* in absolute value, the contributions from the *K* ≠ *A* terms become negligible, and the OQS-PCM­(ω) Hamiltonian approaches the self-consistent field solvation regime. In practice, this requires $E_{A}^{\prime }\approx {\mathrm{E̅}}_{A}$ and $\left|E_{A}^{\prime }-{\mathrm{E̅}}_{K}\right|\gg \omega_{solv}$ for every *A* and *K* ≠ *A*, where ω_solv_ identifies a characteristic frequency marking the tail of the solvent electronic response.

Hereafter, we refer to the approximations presented in eqs 15, 17, and 18 as “cutoff”, “BO-sol” and “SS-pol”, respectively.

## 3 OQS-PCM(ω) Implementation

We have implemented OQS-PCM­(ω), along with its approximate variants, in the Python software package OQSolv,^22^ interfaced with TDPlas^23^ and a locally modified version of GAMESS-US.^24^ Here, we summarize the main features of this implementation and refer the reader to the Supporting Information for further details.

To obtain the solvated states within OQS-PCM­(ω), we require the PCM response matrix Q(ω) , which depends on the PCM cavity shape and tessellation, as well as on the frequency-dependent dielectric function ϵ­(ω).^1^ This matrix can be expressed as^1,5,25^

21 $$Q\left(\omega \right)=-S^{-1}{\left(2\pi \frac{\epsilon \left(\omega \right)+1}{\epsilon \left(\omega \right)-1}I-DA\right)}^{-1}\left(2\pi I-DA\right)$$

where I is the identity matrix, A is the diagonal matrix containing the areas of the cavity tesserae, and S and D are the Calderon BEM matrices. All of these matrices have *N*_tess_ × *N*_tess_ dimensions with *N*_tess_ being the number of tesserae in the cavity tessellation. Since *N*_tess_ might be large, an efficient construction and application of Q(ω) is essential. To this end, we exploit the factorization of Q(ω) introduced in ref 5

22 $$Q\left(\omega \right)=S^{-1/2}TK\left(\omega \right)T^{†}S^{-1/2}$$

23 $$K\left(\omega \right)=-{\left(2\pi \frac{\epsilon \left(\omega \right)+1}{\epsilon \left(\omega \right)-1}I-\Lambda \right)}^{-1}\left(2\pi I-\Lambda \right)$$

where Λ and T are, respectively, the eigenvalue and eigenvector matrix associated with the ancillary matrix $S^{-1/2}DAS^{1/2}$ , and $T^{†}$ denotes the transpose of T .

The representation of eqs 22 and 23 allows us replace the full matrix Q(ω) by its diagonal kernel, K(ω) , in the OQS-PCM Hamiltonian [Disp-formula eq13], provided that the molecular potential matrix elements are transformed according to

24 $$V_{IK,i}=\sum_{j,k}T_{ij}^{†}S_{jk}^{-1/2}\left\langle {\mathrm{\Phi ̅}}_{I}\left|{\mathrm{V̂}}_{k}\right|{\mathrm{\Phi ̅}}_{K}\right\rangle$$

Matrix elements of $T^{†}$ and $S^{-1/2}$ are denoted $T_{ij}^{†}$ and $S_{ij}^{-1/2}$ , respectively. Note that [Disp-formula eq24] must be performed for each tessera and for every braket pair of the $|{\mathrm{\Phi ̅}}_{K}\rangle$ basis. In this way, the computationally demanding inversion and matrix-vector multiplication—scaling as $O\left(N_{tess}^{3}\right)$ —are reduced to the inexpensive evaluation of a diagonal and a Hadamard product, scaling as $O\left(N_{tess}\right)$ . According to this protocol, the role of $Q_{d}$ in the cutoff, BO-sol and SS-pol approximations is played by

25 $$K_{d}=-{\left(2\pi \frac{\epsilon_{d}+1}{\epsilon_{d}-1}I-\Lambda \right)}^{-1}\left(2\pi I-\Lambda \right)$$

Matrices S , D and A are obtained from TDPlas, whereas Λ, $T^{†}S^{-1/2}$ , and the transformation [Disp-formula eq24] are computed in preparatory routines executed prior to the main OQS-PCM­(ω) module.

Having access to kernel matrix elements of *K*_*ii*_(ω) and *K*_d,*ii*_ as well as to the transformed potentials *V*_*IK*,*i*_, we are able to build the OQS-PCM­(ω) Hamiltonian [Disp-formula eq13] or any of its approximated counterparts in [Disp-formula eq15], 17 and 18. In the case of BO-sol, where $K\left(\omega \right)=K_{d}$ , we only need to diagonalize eq 17 once to obtained all of the desired solvated energies and states. By constrast, the cutoff and SS-pol approximations lead to state-specific problems and therefore, require a separate diagonalization of the Hamiltonians in eqs 15 and 18, respectively, for each solvated state. Since the solvation corrections may alter the ordering of the states at each diagonalization, we identify and track the targeted solvated state by its maximum projection onto the corresponding state of the $|{\mathrm{\Phi ̅}}_{A}\rangle$ basis.

OQS-PCM­(ω) Hamiltonian [Disp-formula eq13] is likewise state-specific. As in the cutoff and SS-pol cases, a distinct Hamiltonian must be constructed and diagonalized for each solvated state, and the correct solvated state must again be selected by projection onto the corresponding state of the $|{\mathrm{\Phi ̅}}_{A}\rangle$ basis. However, the full-frequency formulation introduces an additional complication: for every state, the solvated energy appears explicitly within the Hamiltonian to be diagonalized. Consequently, solvated energies are obtained via a self-consistent procedure, starting from the reference energy ${\mathrm{E̅}}_{A}$ of $|{\mathrm{\Phi ̅}}_{A}\rangle$ and iteratively updating it until convergence (see Section S2.5 in the Supporting Information). The workflow we follow is illustrated in Figure 2. The algorithms for OQS-PCM­(ω), cutoff, BO-sol, and SS-pol are implemented in the in-house code OQSolv.^22^

**Figure 2:**
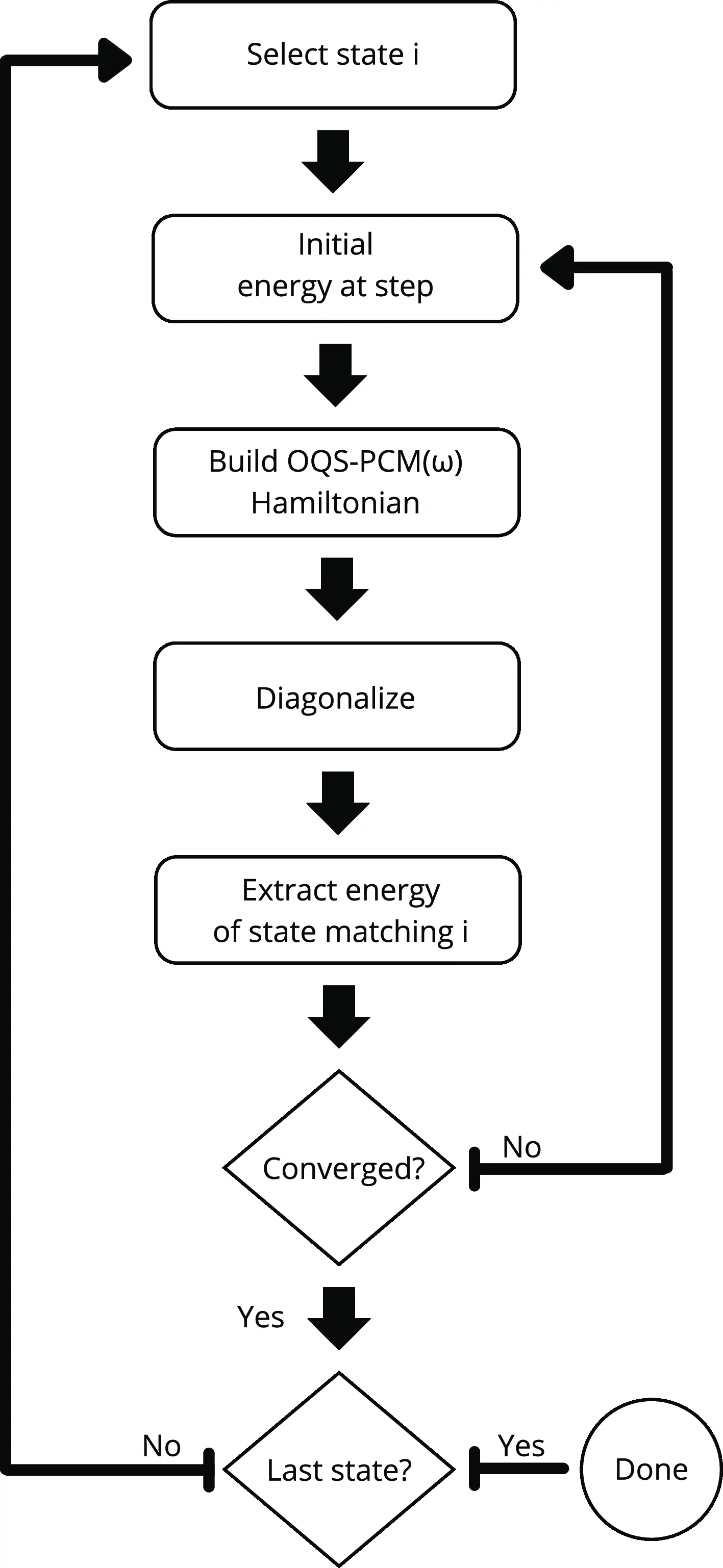
Workflow diagram illustrating the main steps of OQS-PCM­(ω).

In practice, solvated energies *E*_*A*_^′^ are typically close to the basis eigenvalues ${\mathrm{E̅}}_{A}$ , and the self-consistent procedure introduces only negligible corrections. As a useful benchmark, consider the cutoff approach (see Section 2.1), which incorporates the same types of state-specific solute–solvent interactions as OQS-PCM­(ω)—namely polarization, dispersion, and dissipation—while differing in its lack of self-consistency and in its treatment of the frequency dependence of the response function. The dominant deviation between the cutoff and OQS-PCM­(ω) approaches therefore arises from the inclusion of the proper frequency dependence of the response kernel, rather than from the more accurate handling of the Hamiltonian nonlinearity. Whereas in the cutoff method the Hamiltonian [Disp-formula eq13] is constructed for each state using the approximate kernel

26 $$K\left(E_{A}^{\prime }-E_{K}\right)=K_{d}\Theta \left(\omega_{cutoff}-\left|{\mathrm{E̅}}_{A}-E_{K}\right|\right)$$

in OQS-PCM­(ω) the right-hand side is evaluated directly from the empirical frequency dependence of ϵ­(ω) (see Section S2.4 in the Supporting Information).

## 4 Computational Details

Our prototypical systems consist of three molecules in two solvents. The studied solutes are formaldehyde and nitrous acid—both moderately polar—and hydrochloric acid—a strongly polar molecule—(see Figure 3), while the chosen solvents are water and benzene, which differ greatly in polarity. This setup allows us to cover distinct chemical systems characterized by different solute–solvent polarities.

**Figure 3:**
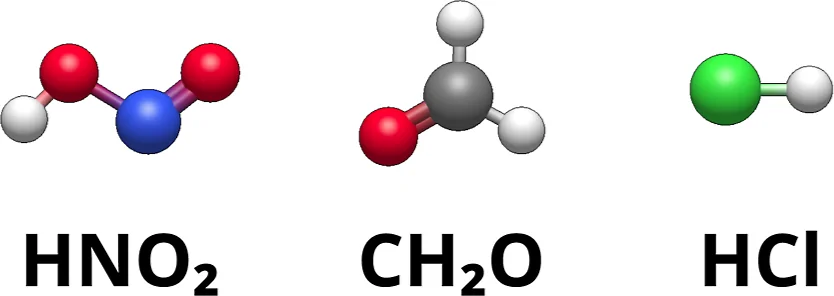
Schematic representations of the molecules chosen as test solutes, namely nitrous acid (HNO_2_), formaldehyde (CH_2_O), and hydrochloric acid (HCl).

Solute ground-state geometries are optimized in Gaussian (v. 16)^26^ using the MP2/6-31 + G­(d,p) level of theory. The starting geometries for these optimizations are taken from the PubChem database,^27^ and are subsequently relaxed in Avogadro v. 1.99.0 ^28,29^ using the MMFF94 force field.

Our OQS-PCM­(ω) calculations are based on the solution of a frozen-solvent problem (see Sections S1.2 and S1.3 in the Supporting Information). To solve the frozen-solvent problem, we perform linear-response time-dependent density functional theory (LR-TDDFT) calculations at the LC-ξBPBE/6-31 + G­(d,p) level (with ξ = 0.33) coupled with PCM within a locally modified^30^ version of GAMESS-US^24^ (forked from v. 2014 R1). In the LR-TDDFT computations, the full space of singlet excited states supported by the atomic orbital basis is requested for each molecule. Using the 6-31 + G­(d,p) basis set, this amounts to *N* = 171, 320, and 600 excited states for hydrochloric acid, formaldehyde, and nitrous acid, respectively.

All calculations are carried out within the integral equation formalism (IEF) implementation of PCM. In practice, the modified version of GAMESS-US reads the static and dynamic IEF-PCM matrices, as well as the cavity tessellation, from files generated by an external code, namely TDPlas.^23^ The molecular cavity is constructed from interlocking spheres centered on each atom of the solute using the GEPOL algorithm.^31^ Cavity sphere radii are taken from default PCM calculations in Gaussian v. 16, matching the van der Waals radii of the atomic species at the sphere centers, scaled by a factor of 1.1. Frozen-solvent approach effectively corresponds to zeroing the dynamic PCM response matrix (or, equivalently, setting the dynamic value of the dielectric constant to 1), while maintaining the appropriate static PCM response for the chosen solvent and molecular cavity.

The modified implementation of GAMESS-US allows us to extract matrix elements of the molecular electrostatic potential between any pair of states, ground or excited, rather than being limited to ground-to-excited transition potentials, evaluated at each cavity tessera (see ref 32). Excitation energies, together with static and transition molecular potentials, are then used to diagonalize the inertial-solvent Hamiltonian (see Sections S1.2 and S2 in the Supporting Information). The resulting eigenstates provide the basis for diagonalizing the OQS-PCM­(ω) Hamiltonian and its approximations introduced above, where a dynamic solvent response is incorporated.

## 5 Results

### 5.1 Polarization, Dispersion and Dissipation Effects

We first evaluate the relative strength of polarization, dispersion, and dissipation contributions to OQS-PCM­(ω) Hamiltonian [Disp-formula eq13]. To that end, in Figure 4-left, we examine the low-lying singlet free energy spectrum of nitrous acid in water, as obtained from progressively more complex models. Polarization-only treatments, either based on the frequency-averaged response (SS-pol­[ϵ_d_]) or on the full dielectric function (SS-pol­[ϵ­(ω)]), yield practically identical spectra, indicating that the explicit frequency dependence of ϵ­(ω) is not critical at this level. Inclusion of dispersion on top of SS-pol­[ϵ­(ω)] (here termed SS-pol + disp­[ϵ­(ω)]) produces sizable energy redshifts of ∼0.17–0.91 eV, depending on the state. It can also produce a reordering of the energy levels, as evidenced by the swapping of the ninth and 10th states in Figure 4 (left panel). Dissipation effects are negligible for the present solute–solvent system, as apparent from the comparison between SS-pol + disp­[ϵ­(ω)] and OQS-PCM­(ω) results. Therefore, dispersion is the dominant contribution to the OQS-PCM­(ω) beyond the standard SS-pol­[ϵ_d_] approach.

**Figure 4:**
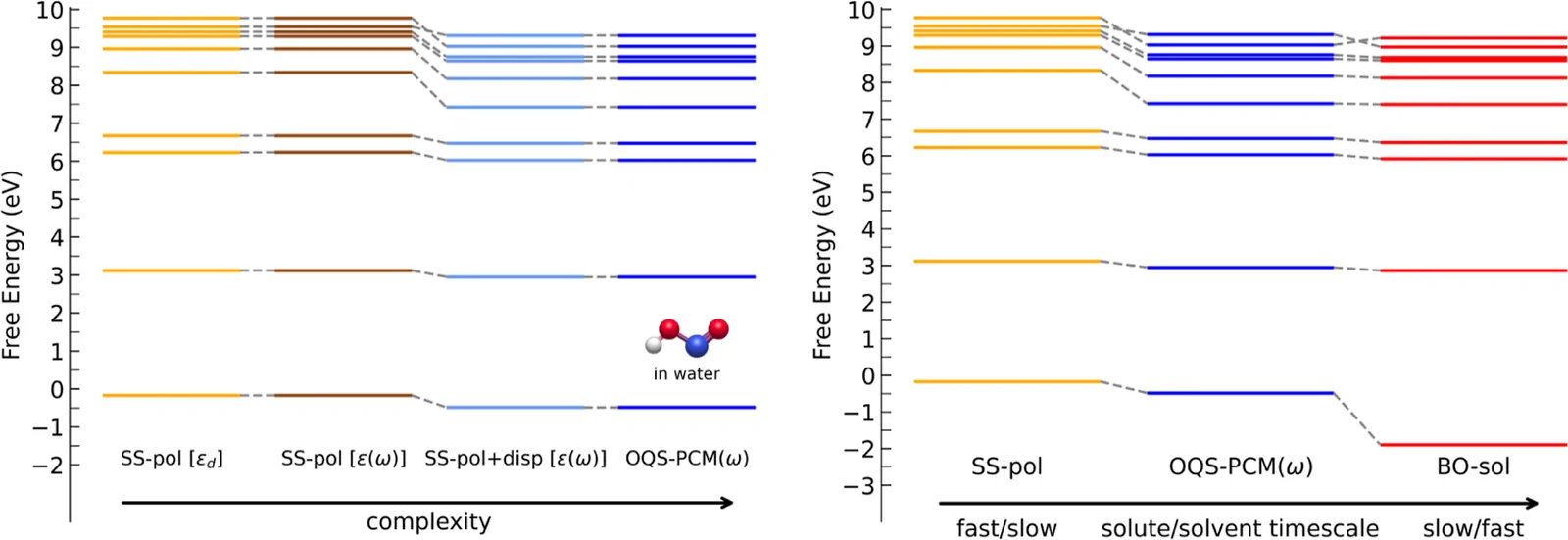
Low-lying singlet free-energy spectrum of nitrous acid in water. Left panel shows results for “SS-pol­[ϵ_d_]”, “SS-pol­[ϵ­(ω)]”, “SS-pol + disp­[ϵ­(ω)]” and OQS-PCM­(ω) approaches. Models are ordered along the *x*-axis in order of increasing complexity. Right panel replots results for SS-pol (same as “SS-pol­[ϵ_d_]”) and OQS-PCM­(ω), and additionally includes BO-sol results. Conceptually, the *x*-axis spans the solute–solvent time scales, ranging from the fast/slow (SS-pol) to the slow/fast (BO-sol) regimes. Only the lowest ten states are shown in both panels. Hamiltonians were diagonalized using 50 states with a resolution of the identity spanning all singlet states under the 6-31 + G­(d,p) basis set, namely *N* = 601.

We probe state-specific self-consistency by comparing first-iteration and converged energies across all frequency-dependent models, including SS-pol­[ϵ­(ω)], SS-pol + disp­[ϵ­(ω)] and OQS-PCM­(ω). As a result of state-specific self-consistency, the maximum energy correction across the first ten states is ∼0.00 and ∼0.02 eV for the simplified models and ∼0.03 eV for the OQS-PCM­(ω), indicating that iterative refinements have only a minor role in energy estimations.

### 5.2 Solute–Solvent Limiting Time Scales: SS-pol and BO-sol

Here, we assess the full-frequency OQS-PCM against its limiting approximations—namely, BO-sol and SS-pol—which arise when solute and solvent electrons are characterized by different time scales. Figure 4 (right panel) illustrates the impact of these assumptions on the free-energy spectra for SS-pol, BO-sol and OQS-PCM­(ω).

First, notice that OQS-PCM­(ω) is not a simple average of SS-pol and BO-sol results. Moreover, we observe that the position of the OQS-PCM­(ω) spectrum relative to the limiting regimes is state-dependent. Indeed, for the ground state, OQS-PCM­(ω) energy is much closer to SS-pol than to the corresponding BO-sol energy, whereas, e.g., for the fifth excited state the situation is reversed. The ground-state solute–solvent dispersion, as estimated from the OQS-PCM­(ω)-to-SS-pol energy shift, amounts to approximately 0.3 eV, and it is overestimated by about 1.4 eV in BO-sol.

The BO-sol approximation includes polarization and dispersion contributions obtained from a frequency-averaged response. In terms of the classification introduced in the previous subsection, the BO-sol approximation corresponds to SS-pol + disp­[ϵ_d_]. The discrepancies between BO-sol and OQS-PCM­(ω) therefore highlight the importance of incorporating the frequency dependence of the solvent dielectric response to accurately account for dispersion. Remarkably, including the frequency-dependent solvent response may impact the energy level rearrangement, as occurs in the case of the ninth and 10th energy levels in Figure 4. We stress that the swap of these levels in Figure 4-left cannot be due to the inclusion of dispersion effects, which are also introduced, however approximately, in BO-sol (or SS-pol + disp­[ϵ_d_]).

As discussed in Sections 2.1.2 and 2.1.3, BO-sol is approximately obtained when all $\left|E_{A}^{\prime }-{\mathrm{E̅}}_{K}\right|$ are much smaller than any solvent electronic excitation energy, whereas SS-pol is approximately obtained when all $\left|E_{A}^{\prime }-{\mathrm{E̅}}_{K}\right|$ , with *A* ≠ *K*, are much larger [see eqs 13]. Although these formal validity conditions are defined globally by the complete set of energy differences entering the OQS-PCM­(ω) response functions, the degree to which each condition is violated may vary from state to state. Across the states shown in Figure 4, the fraction of transitions lying above the onset of the solvent electronic response decreases from approximately 99% to 91%, making the BO-sol condition progressively less severely violated, whereas the fraction lying below the tail of the solvent electronic response increases from approximately 4% to 16%, making the SS-pol condition progressively less well satisfied. This is consistent with the observed tendency of OQS-PCM­(ω) energies to become closer to BO-sol and farther from SS-pol for higher-lying states.

Our OQS-PCM­(ω) framework enables us to include full-frequency polarization, dispersion and dissipation effects in any property derivable from the solvated states, not just the energy spectrum. In particular, we can compute the oscillator strength from the transition energies and dipoles.

Figure 5 (top left panel) shows the absorption spectrum of nitrous acid in water obtained from SS-pol, OQS-PCM­(ω) and BO-sol approaches. Note that dispersion corrections are not only quantitatively but also qualitatively different depending on the state. For instance, the excitation around 6.40 eV (A) is slightly blue-shifted (∼0.11 eV) from SS-pol to OQS-PCM­(ω), whereas the one near 9.46 eV (D) gets red-shifted by 0.33 eV. The oscillator strength changes from SS-pol to OQS-PCM­(ω) for the latter excitations is partly due to a change in the transition dipole moments involved. Indeed, the absolute value of the transition dipole moment diminishes by about 4% for A and 0.2% for D.

**Figure 5:**
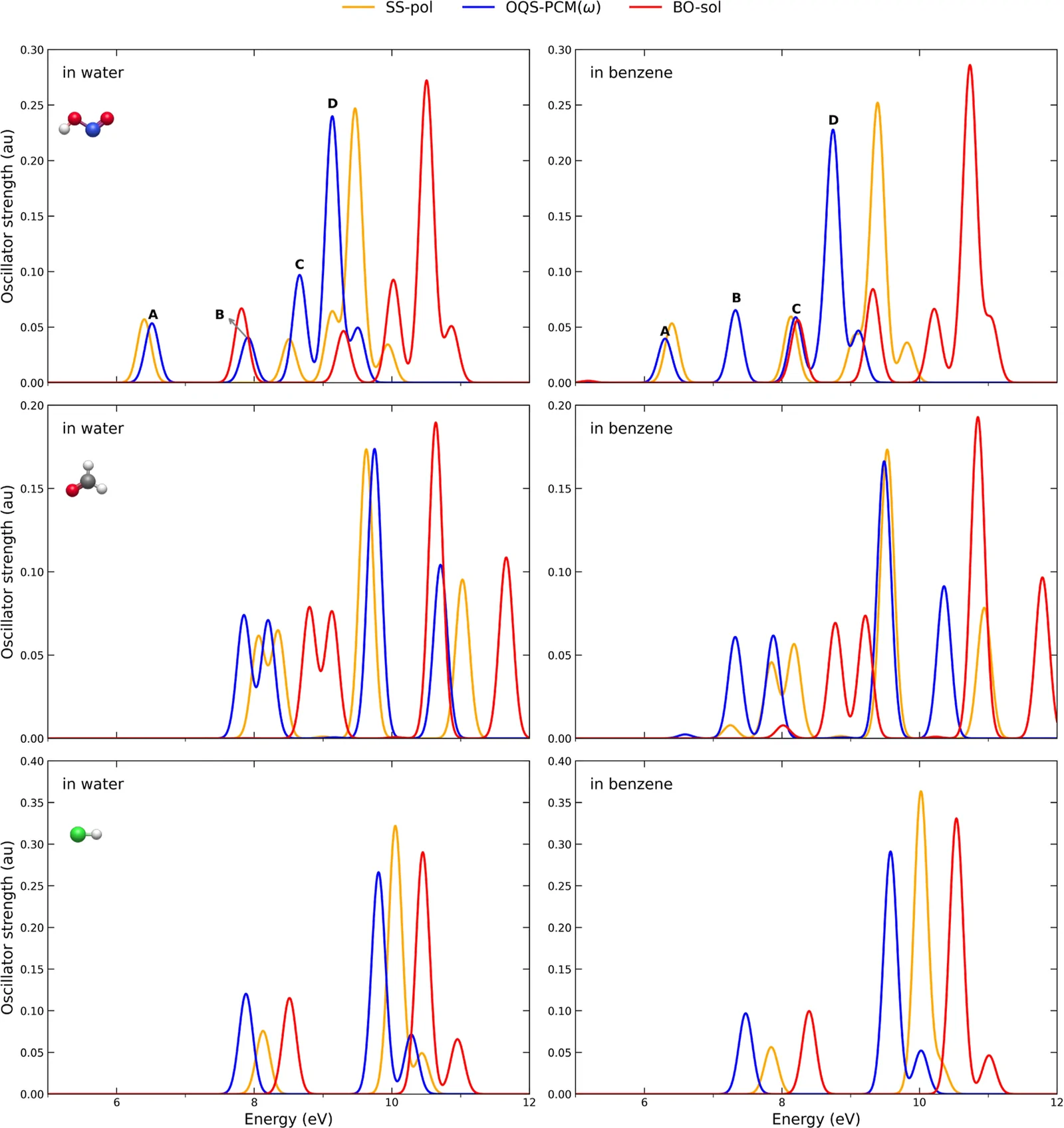
UV absorption spectrum of HNO_2_ (top), CH_2_O (middle) and HCl (bottom) in water (left) and benzene (right) obtained using the SS-pol, BO-sol, and OQS-PCM­(ω) approaches. A–D excitations of HNO_2_ which are referenced in the text are marked for the OQS-PCM­(ω) peaks in the top panels. In all cases, spectra are constructed by convoluting Gaussian functions centered at the transition energies, with heights determined by the corresponding oscillator strengths. The Gaussian broadening is set to 0.1 eV. See Figure 4 for additional details.

Moreover, we observe that OQS-PCM­(ω) results are generally closer to SS-pol than to BO-sol, with corrections of about −0.19 eV on average. On the other hand, BO-sol systematically overestimates excitation energies, by 1.34 eV in average. This is mainly due to the misrepresentation of the dispersion corrections to the ground state [see Figure 4 right].

The pronounced overstabilization of ground-state energies and the corresponding overestimation of excitation energies apparent in BO-sol results, and shown in Figures 4 and 5, respectively, ultimately originate from applying the approach beyond its validity regime. Indeed, the BO-sol condition discussed above is generally not fulfilled in realistic molecular systems, where solute and solvent electronic transitions may overlap. For example, the first UV peak of the water response occurs at approximately 8.15 eV, whereas the fifth electronic excitation of HNO_2_ lies at about 8.66 eV. As a consequence, BO-sol is unreliable as a description of solute–solvent dispersion effects. Because OQS-PCM­(ω) retains the full complex frequency-dependent dielectric response of the solvent, it explicitly accounts for the overlap between the solute electronic transitions and the solvent excitation spectrum, thereby providing an accurate description of solute–solvent dispersion effects within the present theoretical framework.

### 5.3 Full vs Approximate Frequency Dependencies

Previously, we introduced the cutoff approximation, which does not fall into either of the aforementioned limiting regimes. This approximation assumes that the solvent responds only to certain solute *electronic* excitations—specifically, those below the threshold of solvent *electronic* excitations—and that this response is independent of their associated frequency (see eq 14). Given that a full-frequency description of the solvent dielectric function is not always available and that the OQS-PCM­(ω) approach may be computationally demanding, it is worth investigating whether the cutoff approximation can serve as an inexpensive and straightforward alternative. Note that the cutoff approximation depends only on three parameters, which are the ϵ_0_, ϵ_d_ and ω_cutoff_.

In Figure 6 (top left panel), we plot two cutoff approximations to the absorption spectra of nitrous acid in water, along with SS-pol and OQS-PCM­(ω) results. In the first approximation (denoted “cutoff”), ϵ_d_ corresponds to the average of the real part of ϵ­(ω) in the optical range, while ω_cutoff_ is taken as the frequency of the first peak of its imaginary part in the UV range. The second approximation (denoted “cutoff fit”) fits the real part of the dielectric function from 0 to 100 eV (using a uniform step of 10^–4^ eV) to ϵ_d_Θ­(ω_cutoff_ – ω) + Θ­(ω – ω_cutoff_). In practice, we replace the Heaviside step functions Θ­(*x*) by smooth logistic sigmoids *L*(β*x*) during the fit, and take the limit β → ∞ to recover the Heaviside-based expression. Values of ϵ_d_ and ω_cutoff_ for both cutoff approximations are reported in Table 1. Notice that this approximation recovers the proper solvent transparency limit at high frequency, i.e. ϵ­(ω) → 1 for ω → ∞. It is apparent that the cutoff approximation systematically underestimates solvated excitation energies by ∼0.21 eV in average (as compared with OQS-PCM). In fact, cutoff does not reproduce the qualitative state-specific trends of dispersion contributions: for excitation A it predicts a red-shift relative to SS-pol, whereas the OQS-PCM­(ω) result exhibit a slight blue-shift instead. The cutoff fit recovers dispersion effects missing in the (unfitted) cutoff. Indeed, absorption spectra computed from cutoff fit and OQS-PCM­(ω) are generally shifted by ∼−0.05 eV.

**Figure 6:**
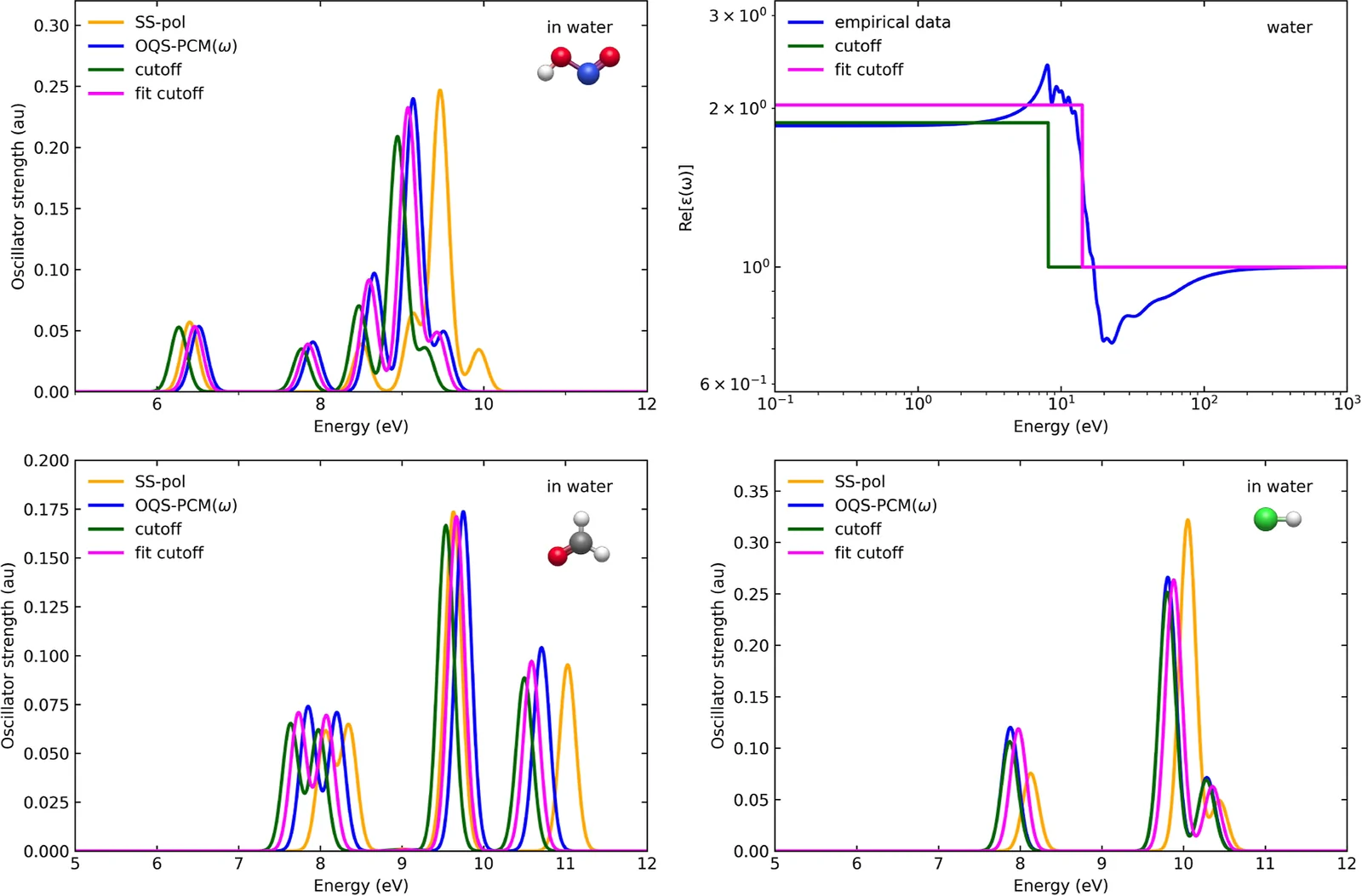
Top left and bottom panels: UV absorption spectrum of HNO_2_ (top left), CH_2_O (bottom left) and HCl (bottom right) in water obtained using the OQS-PCM­(ω), SS-pol, and cutoff approaches. Top right panel: real part of the empirical dielectric function ϵ­(ω) for water [used in OQS-PCM­(ω)], shown together with the two cutoff approximations employed in panel left, plotted on a log–log scale.

**Table 1:** Values of Parameters Entering in the Cutoff Approximations to OQS-PCM­(ω)

	ϵ_d_	ω_cutoff_ (eV)
cutoff	1.88	8.15
fitted cutoff	2.03	14.07

To explain these results, we turn to the frequency dependence of the dielectric function. In Figure 6 (top right panel) the real part of the dielectric function is plotted against energy, together with its cutoff approximations, on a log–log scale. Remarkably, the cutoff *simpliciter* reproduces the zero- and infinite-frequency limits of the real part of the dielectric function quite well, while the cutoff fit correctly captures the frequency at which the real part of ϵ­(ω) drops from its zero-frequency value toward its infinite-frequency limit (i.e., 1). Both descriptions misrepresent the intermediate region from 3 to 200 eV, which includes ∼69% of the valence excitations of nitrous acid. In particular, details of the solvent response in the 3–14 eV range are completely lost. Since both cutoffs deviate similarly from the empirical dielectric profile at energies above 14 eV, the key difference between these approximations [see Figure 6 top left] lies in how accurately they capture the dielectric response in the 3–14 eV range, where the first 30 solute excitations occur. Even though the fitted cutoff trend cannot reproduce the spikes in this energy region, it is on average closer to the empirical values than the (unfitted) cutoff. This is also the case for the other test solutes, namely formaldehyde and hydrochloric acid, as shown in Figure 6 bottom panels.

### 5.4 Solvent Polarity

In this section, we analyze the effect of solvent polarity on the excitation energies and absorption spectra by switching from a strongly polar solvent such as water (ϵ_0_ = 78.74) to a weakly polar solvent such as benzene (ϵ_0_ = 2.24). The top right panel of Figure 5 shows results analogous to those discussed for the top-left panel, now for nitrous acid in liquid benzene.

With respect to water [Figure 5, top left], the case of benzene [Figure 5, top right] shows a relative quenching of absorption bands A, C, and D, and a relative enhancement of band B (see Figure 5). The solvatochromic shifts of the A–D bands, as calculated with OQS-PCM­(ω), differ between the two solvents by −0.21, −0.58, −0.46, and −0.56 eV, respectively. These results confirm that spectral shifts are both state-specific and solvent-dependent.

In contrast, SS-pol predicts differences of −0.00, −0.38, −0.08, and −0.20 eV for the A–D excitations, respectively. This comparison suggests that the solvatochromism of bands A, C, and D is largely dominated by dispersion effects, with band A exhibiting an almost purely dispersive contribution. Even for band B, dispersion accounts for more than half of the total shift difference relative to polarization.

Remarkably, in this case, dispersion (−0.21, −0.20, −0.38, and −0.36 eV for A–D) and polarization contributions to solvatochromism are qualitatively anticorrelated: dispersion tends to be larger when polarization is smaller, and vice versa. However, the corresponding state-specific variations are not quantitatively correlated, indicating that the two contributions follow independent trends and one cannot be inferred from the other. These observations emphasize the nontrivial role of state-specific dispersion in accurately reproducing solvatochromic effects.

Furthermore, we find that even in a weakly polar solvent such as benzene, the BO-sol approach systematically and significantly overestimates excitation energies [see Figure 5, top right]. This BO-sol deficiency is also observed for the other test solutes, namely formaldehyde and hydrochloric acid, both in water and benzene (see the middle and bottom panels of Figure 5).

### 5.5 Experimental Validation

Finally, we benchmark our results for nitrous acid (in water and benzene), formaldehyde (in water), and hydrochloric acid (in water) against experimental data.^33–35^ Table 2 shows the excitation energy of the first optical transition (S0 → S1) of these systems for the BO-sol, SS-pol, and OQS-PCM­(ω) approaches.

**Table 2:** S0 → S1 Excitation Energy (eV) of Test Molecules in Solution as Computed with the SS-pol, BO-sol, and OQS-PCM­(ω) Approaches Including Vibronic Effects^a^

system	SS-pol	BO-sol	OQS-PCM(ω)	exp.
HNO_2_ in water	3.23	4.70	3.36	3.33
HNO_2_ in benzene	3.17	5.09	3.16	3.46
CH_2_O in water	3.73	4.77	3.88	4.26
HCl in water	8.07	8.46	7.80	7.91

a The last column reports the experimental (exp.) values from ref 33 for HNO_2_, ref 34 for CH_2_O, and ref 35 for HCl. In all cases, the reported excitation energy corresponds to the highest-intensity vibrational band of the optical transition. Details on the inclusion of vibronic and other effects are provided in the Supporting Information.

Since the test molecules are small, they are significantly affected by nuclear motion. Therefore, in each of the previous methodologies, we include vibronic effects along the lines detailed in Section S2.6 of the Supporting Information. Vibronic corrections to the solvated electronic excitation energies are computed from standard-PCM electronic-structure calculations under the harmonic potential-energy-surface approximation. Depending on the system, this involves the Franck–Condon or Herzberg–Teller approximations together with either the adiabatic or vertical Hessian models, following established methodologies.^36,37^ Introducing vibronic effects—albeit approximately—is critical for a physically meaningful comparison between the solvation models. In purely electronic spectra, the SS-pol approach may appear closer to experiment than OQS-PCM­(ω), due to the missing shift associated with a combination of zero-point vibrational energies and the energetic location of the Franck–Condon envelope maximum.

Additionally, we incorporate into OQS-PCM­(ω) the LR-PCM^38–40^ correction as a proxy for the dispersion contribution arising from the solute response to solvent fluctuations, as recommended in the cLR^2^ approach.^41^ For the S0 → S1 of our prototypical systems, this correction is negligible with respect of the state-specific dispersion contribution naturally contained within OQS-PCM­(ω). See Section S2.7 in the Supporting Information for more details.

For all systems, OQS-PCM­(ω) results are the closest to the experimental values, with a mean signed error across systems of −0.135 eV. Except for the case of nitrous acid in water, which closely matches the experiment (+0.03 eV), OQS-PCM­(ω) slightly underestimates the experimental excitation energies, with the largest deviation occurring for formaldehyde in water (−0.38 eV). The second closest approach is SS-pol, with a mean signed error across systems of −0.190 eV. If we estimate the sign of the net dispersion effects from the differences between the experimental and SS-pol values, we find that OQS-PCM­(ω) consistently follows the expected trend for systems in water: positive dispersion contributions for nitrous acid and for formaldehyde, and negative dispersion for hydrochloric acid. (The case of nitrous acid in benzene is special, as state-specific and linear-response dispersion effects nearly cancel each other.) This pattern is not followed by BO-sol, which systematically overestimates the experimental excitation energies and exhibits a bias toward a positive effect of dispersion. In the case of nitrous acid, BO-sol overestimates the excitation energy by more than 1 eV, consistent with our previous analyses. However, for formaldehyde and hydrochloric acid in water, the BO-sol errors remain sizable—0.51 and 0.55 eV, respectively. The remaining quantitative differences between OQS-PCM­(ω) and experimental values are likely due to the underlying electronic structure theory, especially the choice of the exchange–correlation functional with known errors in the range of 0.2–0.3 eV.

These results confirm the importance of accounting for the frequency-dependent solvent response and point to OQS-PCM­(ω) as the most accurate method, while reiterating that BO-sol is not a realistic approximation for incorporating solvation effects in absorption spectra.

### 5.6 Convergence with the Number of Excited States

Our OQS-PCM­(ω) implementation is based on a resolution-of-the-identity (RI) representation, expressed as a sum over multiparticle electronic excited states of the solute. This formulation has been shown to exhibit slow convergence with respect to the number of excited states, particularly in the case of BO-sol, as reported in ref 18.

To further analyze this behavior, Figure 7 reports the ground-state (GS) solvation energy as a function of the total number of excited states included in the RI. Nitrous acid in water is taken as a prototypical system, while results for additional solute–solvent combinations are provided in the Supporting Information.

**Figure 7:**
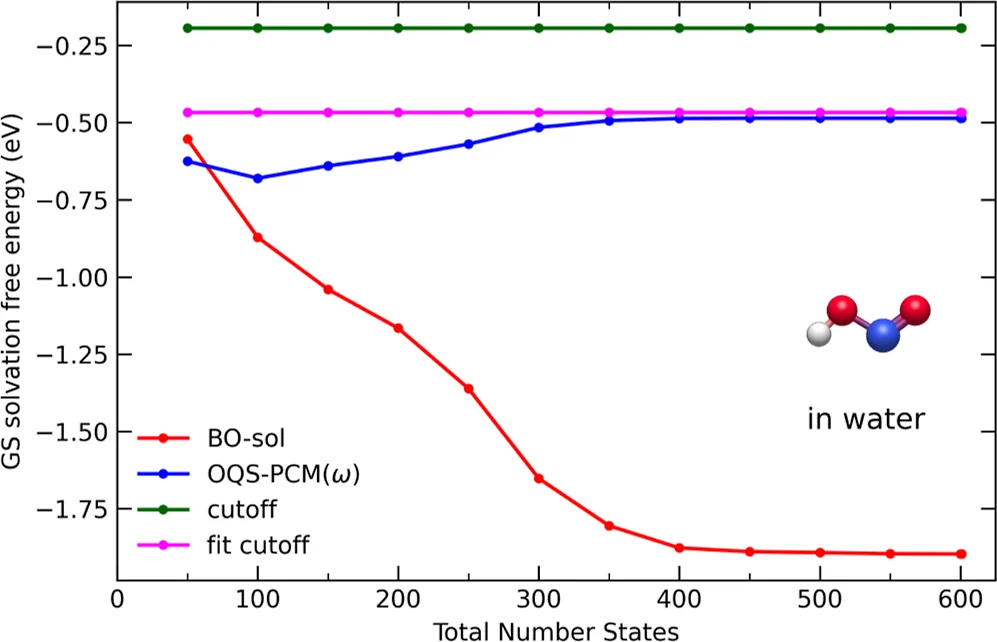
GS solvation free energy as a function of the total number of excited states included in the resolution of the identity for nitrous acid in water. Results for OQS-PCM­(ω), BO-sol, and the cutoff approaches are plotted in blue, red, dark green and magenta, respectively. The *x* axis is sampled in increments of 50 states and includes the point corresponding to the full space of singly excited singlet states (601 states for HNO_2_ with the 6-31 + G­(d,p) basis set).

First, we observe that for all approaches, the GS solvation free energy converges as the number of excited states included in the RI increases. However, the convergence profiles differ markedly among the methods. Cutoff and fitted cutoff results are already converged from the first data point (50 states), whereas both BO-sol and OQS-PCM­(ω) exhibit slow convergence with respect to the size of the RI space. In particular, BO-sol displays a monotonically decreasing trend, while OQS-PCM­(ω) shows a nonmonotonic behavior, characterized by an initial decrease (up to ∼100 states) followed by a gradual increase toward the corresponding fitted-cutoff value.

The early convergence of cutoff approaches is expected since, by construction, excited states *K* with $\left|{\mathrm{E̅}}_{0}-{\mathrm{E̅}}_{K}\right|>\omega_{cutoff}$ do not contribute [see eq 15]. In contrast, the results of BO-sol and OQS-PCM­(ω) change upon the inclusion of each additional state in the RI. In particular, for BO-sol, the contribution of each additional state to the solvation energy is always negative, whereas for OQS-PCM­(ω) it can be either negative or positive depending on the value of ϵ­(ω).

This behavior can be understood in terms of a simpler continuum solvation model, where the solute is treated as a point dipole and the solvent-excluded cavity is spherical (see the OQS-Onsager model in the Supporting Information). In this framework, each term in the RI is weighted by a frequency-dependent factor $-g\left({\mathrm{\omega ̅}}_{0^{\prime }K}\right)\propto -\left(\epsilon \left({\mathrm{\omega ̅}}_{0^{\prime }K}\right)-1\right)/\left(2\epsilon \left({\mathrm{\omega ̅}}_{0^{\prime }K}\right)+1\right)$ . In BO-sol, this factor is frequency-independent and negative, since $\epsilon \left({\mathrm{\omega ̅}}_{0^{\prime }K}\right)=\epsilon_{d}>1$ , explaining the observed monotonic decrease of the GS solvation free energy. In contrast, the convergence pattern of OQS-PCM­(ω) arises from the frequency-dependent dielectric response of the solvent. Indeed, physical dielectric functions obey the high-frequency transparency trend, ϵ­(ω) → 1^–^ as ω → ∞, while typical solvents satisfy ϵ(0) > 1. As a result, $g\left({\mathrm{\omega ̅}}_{0^{\prime }K}\right)$ changes sign at intermediate frequencies, naturally leading to the nonmonotonic behavior of OQS-PCM­(ω) GS solvation free energy as the RI space increases.^42^

Notably, the converged OQS-PCM­(ω) value is close to that obtained with the fitted cutoff approach, highlighting the critical role of reproducing the zero- and high-frequency limits, even in the absence of the full frequency-dependent, complex-valued dielectric function. The weaker GS stabilization of the cutoff approach, compared to the fitted cutoff, is a consequence of the smaller ϵ_d_ used in this calculation (see Table 1). Furthermore, the nonphysical deviation of BO-sol from the converged values of OQS-PCM­(ω) stems from the cumulative stabilization contributed by each term in the RI, which is not modulated by the correct high-frequency response of the solvent (i.e., ϵ­(ω) → 1 as ω → ∞).

Finally, the slow convergence of BO-sol originates from the dependence of the transition potentials $\left\langle {\mathrm{\Phi ̅}}_{0}\left|{\mathrm{V̂}}_{i}\right|{\mathrm{\Phi ̅}}_{K}\right\rangle$ on the excited-state number *K* [see eq 13]. In the case of OQS-PCM­(ω), the reason for the slow convergence is 2-fold: first, the dependence of the transition potentials on *K*, as in BO-sol; and second, the long tail of the real part of the dielectric function as it approaches the high-frequency transparency limit (see Figure 6, right panel), which leads to a slow attenuation of the contribution from high-energy transitions.

Within a RI formulation, the slow convergence problem can only be remedied by including a large fraction of the excited-state manifold (350–400 states in the case of HNO_2_), often exceeding half of the full excitation space. While feasible for small molecular systems such as those considered here, this requirement leads to substantial computational demands for larger molecules. Furthermore, sum-overstates approaches based on DFT are known to significantly overestimate exact molecular polarizabilities for medium-sized systems.^43^ For these reasons, a scalable and quantitatively accurate OQS-PCM­(ω) implementation will ultimately require abandoning the explicit sum-overstates representation, as is commonly done in modern polarizability calculations. Work along these lines is currently in progress. The present results should therefore be regarded as a proof-of-concept application of OQS-PCM­(ω) to organic molecules, highlighting the importance of consistently accounting for the full frequency dependence of the solvent response.

## 6 Concluding Remarks

In the context of modeling solvated molecules, we have introduced a full-frequency implementation of the open quantum system-polarizable continuum model, OQS-PCM­(ω), which incorporates solute–solvent polarization and dispersion interactions, as well as energy dissipation from the solute to the solvent, within a unified framework. This approach enables the computation of state-specific solvation effects that depend on the full frequency-dependent dielectric response of the solvent, rather than only on its static and optical (high-frequency) limits.

OQS-PCM­(ω) is formulated within the general TD-SSE-pol framework, in which the electronic degrees of freedom of the solvent are traced out to yield an effective, non-Markovian electronic dynamics for the solute. In the present full-frequency implementation, we neglect the stochastic component of the dynamics, which accounts for dispersion effects arising from the solute’s response to solvent fluctuations, while retaining the contribution associated with the solvent’s response to solute fluctuations. This approximation is justified for a stationary description, after averaging over environmental fluctuations (see Section S1.1 of the Supporting Information). These assumptions are physically appropriate for macroscopic solutions of weakly interacting solute and solvent molecules, when probed by steady-state spectroscopies. Moreover, the missing solvent-fluctuation dispersion component in OQS-PCM­(ω) excitation energies can be approximately accounted for by including the LR-PCM contribution, as in the cLR^2^ protocol.^41^

In our approach, polarization arising from the inertial (nuclear) degrees of freedom of the solvent is treated as frozen and equilibrated with the ground-state solute. The dynamical polarization associated with the solvent’s faster, electronic degrees of freedom is instead described through its empirical frequency-dependent dielectric function, thereby consistently accounting for the relevant time scales of the solute–solvent interaction.

To illustrate the implications of this formulation, we applied OQS-PCM­(ω) to the calculation of absorption spectra of small molecules in both strongly and weakly polar solvents. Comparisons with the self-consistent and Born–Oppenheimer PCM regimes highlight the importance of accounting for the full range of electronic time scales, beyond the limiting cases of slow or fast solvent response. In particular, OQS-PCM­(ω) yields genuinely state-specific solvation effects, leading to both blue and red shifts of electronic transitions and, consequently, to quantitative and qualitative changes in the estimated dispersion contributions to excited-state energies. Comparison with experimental reference data further reveals a substantial overestimation of dispersion effects by the Born–Oppenheimer solvation approximation, which is not observed in OQS-PCM­(ω), irrespective of the polarity of the solute and solvent. When vibronic effects are properly accounted for, OQS-PCM­(ω) is found to be in good agreement with experiment.

We further find that OQS-PCM­(ω) results can often be reasonably reproduced by a cutoff approach, in which the solvent response is approximated by a step function with an effective frequency range. The close agreement between OQS-PCM­(ω) and cutoff results suggests that several observables, such as absorption spectra, may be relatively insensitive to the detailed frequency-resolved solvent response, provided that the solvent responds correctly on average and that the high-frequency transparency limit is properly enforced. In this sense, the cutoff approach may offer a useful and computationally efficient alternative when a sufficiently detailed empirical dielectric function is not available.

## Supplementary Material



## Data Availability

The data underlying this study are available in the published article, the Supporting Information, and in the Zenodo repository at 10.5281/zenodo.20084781. The Zenodo repository contains representative input and output files for each stage of the computational protocols employed in this work. A README file is also provided to describe the computational workflow and the organization of the repository contents. The main source code, OQSolv, implementing OQS-PCM­(ω) and related approximations, together with a user manual and representative examples, is available in the GitHub repository at https://github.com/LIMElab-theochem/OQSolv. State and transition electrostatic potentials, which constitute key ingredients of OQS-PCM­(ω), were generated using a locally modified version of GAMESS-US^24^ forked from version 2014 R1. Owing to the licensing terms of GAMESS-US, the modified source code cannot be publicly redistributed. The TDPlas code available at https://github.com/stefano-corni/WaveT_TDPlas was employed to generate GEPOL solvent-excluded molecular cavity tessellations, Calderón **S** and **D** matrices, and static and dynamic PCM response matrices. All data required to reproduce the results reported in this work are provided through the repositories described above.

## Notes

The Supporting Information contains, first, an additional theoretical discussion of *the stationary ensemble-averaged limit of the stochastic Schrödinger equation*, *inertial and dynamic polarization*, *frozen-solvent Hamiltonian*, and *OQS-Onsager model*. Next, it provides a detailed description of our computational protocols, including geometry optimizations, frozen-solvent calculations and postprocessing, analytical representations of frequency-dependent dielectric functions, the implementation of the OQS-continuum­(ω) self-consistent algorithm, the inclusion of vibronic effects, and related procedures. We present OQS-Onsager­(ω) results for nitrous acid in water, and discuss their comparison with the corresponding PCM results. Additionally, we provide figures analogous to Figure 4 of the main text for formaldehyde and hydrochloric acid, as well as a comparison of SS-pol, BO-sol, and OQS-PCM­(ω) with the frozen-solvent approximation, LR-PCM, and cLR-PCM for the molecules in the test set, and additional convergence results for nitrous acid in benzene, formaldehyde in water and benzene, and hydrochloric acid in water and benzene. We close with a comparison between OQS-PCM­(ω) and the SMSSP approaches for incorporating dispersion effects ex post^9^ (PDF)
