# Supplementary material for: A Fully Frequency-Dependent Formulation of the Open Quantum System Polarizable Continuum Model
Source: J Chem Theory Comput. 2026 Aug 12;22(16):8466–80. doi: 10.1021/acs.jctc.6c00904 (PMC13523641; doi:10.1021/acs.jctc.6c00904)
Supplement: Supplementary file 1 [file ct6c00904_si_001.pdf]

Supplementary Information for "A fully frequency-dependent formulation of the Open Quantum System Polarizable Continuum Model"

Gabriel Gil,[*,†,‡] Stefano Corni,[¶,§] and Ciro A. Guido[*,†]

†*Dipartimento di Scienze e Innovazione Tecnologica, Università del Piemonte Orientale, Viale T. Michel 11, 15121 Alessandria, Italy*

‡*Instituto de Cibernética, Matemática y Física (ICIMAF), Calle E esquina 15, No. 309, La Habana 10400, Cuba*

¶*Department of Chemical Sciences, University of Padova, via Marzolo 1, 35100, Padova, Italy*

§*CNR Institute of Nanoscience, via Campi 213/A, 41100, Modena, Italy*

E-mail: gabrieljose.gilperez@uniupo.it; ciro.guido@uniupo.it

Contents

S1 Additional Theoretical Discussions **2**

S1.1 Derivation of the stationary ensemble-averaged limit of the stochastic Schrödinger equation . . . 2

S1.2 Inertial and Dynamic Polarization . . . 9

S1.3 Frozen-Solvent Hamiltonian . . . 11

S1.4 OQS-Onsager Model . . . 12

S2 Additional Computational Details **15**

S2.1 Frozen-Solvent Calculations: Onsager Model 15

S2.2 Post-Processing of PCM and Onsager Frozen-Solvent Results: Transition Dipoles and Potentials 15

S2.3 From Frozen-Solvent to the Inertial-Solvent Basis Set 16

S2.4 Frequency-Dependent Dielectric Functions of solvents 16

S2.5 OQS-Continuum(ω) Algorithm 17

S2.6 Electronic and Vibronic Corrections in OQS-PCM Absorption Spectrum Calculations 19

S2.7 Full Dispersion Effects in OQS-PCM 23

S3 Additional Results **24**

S3.1 PCM vs. Onsager Model 24

S3.2 Formaldehyde and Hydrochloric Acid in Water and Benzene 25

S3.3 Comparison of LR, cLR, SS-pol, BO-sol and OQS-PCM(ω) 28

S3.4 Convergence with the Number of Excited States: Other Systems 29

S3.5 Comparison of Solute-Solvent Dispersion with SMSSP Approach 31

References **33**

S1 Additional Theoretical Discussions

S1.1 Derivation of the stationary ensemble-averaged limit of the stochastic Schrödinger equation

In this section, we clarify the relation between the time-dependent stochastic Schrödinger equation for a polarizable continuum bath (TD-SSE-pol) and the stationary OQS-PCM(ω) formulation used in the main text.[1,2] The stationary problem is not defined trajectory by trajectory, because the explicitly time-dependent stochastic fields do not, in general, admit

a strictly stationary solution for an individual realization. Instead, the stationary OQS-PCM(ω) states are obtained by projecting the deterministic equation for the ensemble-averaged coherent amplitude onto the stationary regime.

TD-SSE-pol and perturbative order counting. To make the weak-coupling order explicit, we introduce the usual perturbative parameter λ multiplying the interaction with the dynamical solvent charges, $\hat{\hat{H}}_{SB} \to \lambda \hat{\hat{H}}_{SB}$, while the modified system Hamiltonian $\hat{\hat{H}}_S$, which already contains the mean inertial polarization (as explained in Sec. S1.2), is kept as the zeroth-order Hamiltonian. The physical equation is recovered by setting $\lambda = 1$ at the end. With an initial time t_0, TD-SSE-pol reads

$$\begin{aligned} i\frac{\partial}{\partial t}|\Psi_\alpha(t)\rangle = \hat{\hat{H}}_S|\Psi_\alpha(t)\rangle + \lambda \sum_i \eta_i^\alpha(t)\hat{V}_i|\Psi_\alpha(t)\rangle \\ - i\lambda^2 \int_{t_0}^{t} dt' \sum_{ij} C_{ij}^{\mathrm{sym}}(t-t')\hat{V}_i e^{-i\hat{\hat{H}}_S(t-t')}\hat{V}_j|\Psi_\alpha(t')\rangle \\ + \frac{\lambda^2}{2}\int_{t_0}^{t} dt' \sum_{ij} Q_{ij}(t-t')\hat{V}_i e^{-i\hat{\hat{H}}_S(t-t')}\hat{V}_j|\Psi_\alpha(t')\rangle + \mathcal{O}(\lambda^3). \end{aligned} \tag{S1}$$

Here, α labels a realization of the stochastic process. The limit $t_0 \to -\infty$ gives the stationary-history convention adopted in the main text. It is convenient to define

$$\hat{N}_\alpha(t) \equiv \sum_i \eta_i^\alpha(t)\hat{V}_i, \tag{S2}$$

$$\hat{\mathcal{K}}(\tau) \equiv \sum_{ij}\left[-iC_{ij}^{\mathrm{sym}}(\tau) + \frac{1}{2}Q_{ij}(\tau)\right]\hat{V}_i e^{-i\hat{\hat{H}}_S\tau}\hat{V}_j, \qquad \tau \geq 0. \tag{S3}$$

The Gaspard–Nagaoka stochastic fields are complex Gaussian noise satisfying[1]

$$\overline{\eta_i^\alpha(t)} = 0, \tag{S4}$$

$$\overline{\eta_i^\alpha(t)\eta_j^\alpha(t')} = 0, \tag{S5}$$

$$\overline{[\eta_i^\alpha(t)]^*\eta_j^\alpha(t')} = C_{ij}(t-t'), \qquad C_{ij}(\tau) = C_{ij}^{\mathrm{sym}}(\tau) + \frac{i}{2}Q_{ij}(\tau), \qquad \tau \geq 0. \tag{S6}$$

Ensemble average through second order. We define the ensemble-averaged coherent amplitude as

$$|\Psi_{\mathrm{av}}(t)\rangle \equiv \overline{|\Psi_\alpha(t)\rangle}, \tag{S7}$$

and assume that the initial system state is independent of the stochastic realization. We then expand each trajectory as

$$|\Psi_\alpha(t)\rangle = |\Psi^{(0)}(t)\rangle + \lambda|\Psi_\alpha^{(1)}(t)\rangle + \lambda^2|\Psi_\alpha^{(2)}(t)\rangle + \mathcal{O}(\lambda^3). \tag{S8}$$

At zeroth and first order, Eq. (S1) gives

$$i\frac{\partial}{\partial t}|\Psi^{(0)}(t)\rangle = \hat{\bar{H}}_S|\Psi^{(0)}(t)\rangle, \tag{S9}$$

$$i\frac{\partial}{\partial t}|\Psi_\alpha^{(1)}(t)\rangle = \hat{\bar{H}}_S|\Psi_\alpha^{(1)}(t)\rangle + \hat{N}_\alpha(t)|\Psi^{(0)}(t)\rangle. \tag{S10}$$

Introducing $\hat{U}_0(t) = e^{-i\hat{\bar{H}}_S t}$ and taking $|\Psi_\alpha^{(1)}(t_0)\rangle = 0$, the first-order correction is

$$|\Psi_\alpha^{(1)}(t)\rangle = -i\int_{t_0}^{t} ds\, \hat{U}_0(t-s)\hat{N}_\alpha(s)|\Psi^{(0)}(s)\rangle. \tag{S11}$$

Averaging Eq. (S1) and using the linearity of the deterministic memory kernel gives

$$\begin{aligned} i\frac{\partial}{\partial t}|\Psi_{\mathrm{av}}(t)\rangle = \hat{\bar{H}}_S|\Psi_{\mathrm{av}}(t)\rangle + \lambda\,\overline{\hat{N}_\alpha(t)|\Psi_\alpha(t)\rangle} \\ + \lambda^2\int_{t_0}^{t} dt'\,\hat{\mathcal{K}}(t-t')|\Psi_{\mathrm{av}}(t')\rangle + \mathcal{O}(\lambda^3). \end{aligned} \tag{S12}$$

The stochastic contribution can be expanded as

$$\begin{aligned} \lambda\,\overline{\hat{N}_\alpha(t)|\Psi_\alpha(t)\rangle} = \lambda\,\overline{\hat{N}_\alpha(t)}|\Psi^{(0)}(t)\rangle \\ + \lambda^2\,\overline{\hat{N}_\alpha(t)|\Psi_\alpha^{(1)}(t)\rangle} + \mathcal{O}(\lambda^3). \end{aligned} \tag{S13}$$

The first term on the right-hand side vanishes because $\overline{\eta_i^\alpha(t)} = 0$. Since the zeroth-order

wave function is independent of the stochastic realization, this implies

$$\overline{\hat{N}_\alpha(t)|\Psi^{(0)}(t)\rangle} = \overline{\hat{N}_\alpha(t)}|\Psi^{(0)}(t)\rangle = 0. \tag{S14}$$

Substituting Eq. (S11) into the second term yields

$$\begin{aligned}\overline{\hat{N}_\alpha(t)|\Psi^{(1)}_\alpha(t)\rangle} = & -i\sum_{ij}\int_{t_0}^{t} ds\,\overline{\eta_i^\alpha(t)\eta_j^\alpha(s)} \\ & \times \hat{V}_i\hat{U}_0(t-s)\hat{V}_j|\Psi^{(0)}(s)\rangle = 0,\end{aligned} \tag{S15}$$

where the last equality follows from

$$\overline{\eta_i^\alpha(t)\eta_j^\alpha(s)} = 0. \tag{S16}$$

Consequently,

$$\lambda\,\overline{\hat{N}_\alpha(t)|\Psi_\alpha(t)\rangle} = \mathcal{O}(\lambda^3) \tag{S17}$$

Since TD-SSE-pol is formulated through second order in the coupling, we retain the conservative remainder $\mathcal{O}(\lambda^3)$. The ensemble-averaged coherent amplitude therefore obeys the deterministic equation

$$i\frac{\partial}{\partial t}|\Psi_{\rm av}(t)\rangle = \hat{\bar{H}}_S|\Psi_{\rm av}(t)\rangle + \lambda^2\int_{t_0}^{t} dt'\,\hat{\mathcal{K}}(t-t')|\Psi_{\rm av}(t')\rangle + \mathcal{O}(\lambda^3). \tag{S18}$$

Thus, the explicit stochastic forcing does not contribute to the equation for the ensemble-averaged amplitude through second order in the system–solvent coupling (but do contribute to the first order at each trajectory of course). At this order, all effects of the dynamical solvent degrees of freedom entering the coherent amplitude are contained in the deterministic non-Markovian memory kernel.

We remark that the preceding result is specific to the complex-noise stochastic unraveling

introduced by Gaspard and Nagaoka,[1] and that alternative choices[3] may not lead to the same cancellation of the stochastic contribution to the evolution of the ensemble-averaged amplitude through second order in the system–solvent coupling.

Frequency-domain stationary formulation. Having shown that the explicit stochastic contribution vanishes from the equation for the ensemble-averaged coherent amplitude through second order in the system–solvent coupling, we now seek stationary solutions of Eq. (S18) in the form

$$|\Psi_{\mathrm{av}}(t)\rangle = e^{-itE'_A}|\Phi'_A\rangle. \tag{S19}$$

Substituting Eq. (S19) into Eq. (S18), taking the stationary-history limit $t_0 \to -\infty$, and introducing $\tau = t - t'$, we obtain

$$\begin{aligned} E'_A|\Phi'_A\rangle = \hat{\bar{H}}_S|\Phi'_A\rangle \\ + \lambda^2 \int_0^\infty d\tau\, e^{iE'_A\tau}\hat{\mathcal{K}}(\tau)|\Phi'_A\rangle + \mathcal{O}(\lambda^3). \end{aligned} \tag{S20}$$

Introducing the resolution of the identity in terms of the eigenstates of the modified system Hamiltonian,

$$\hat{I} = \sum_K |\bar{\Phi}_K\rangle\langle\bar{\Phi}_K|, \qquad \hat{\bar{H}}_S|\bar{\Phi}_K\rangle = \bar{E}_K|\bar{\Phi}_K\rangle, \tag{S21}$$

together with the definition of the memory kernel, Eq. (S3), yields

$$\begin{aligned} E'_A|\Phi'_A\rangle = \hat{\bar{H}}_S|\Phi'_A\rangle \\ - i\lambda^2 \sum_K \sum_{ij} \int_0^\infty d\tau\, e^{i\bar{\omega}_{A'K}\tau} C_{ij}^{\mathrm{sym}}(\tau)\hat{V}_i|\bar{\Phi}_K\rangle\langle\bar{\Phi}_K|\hat{V}_j|\Phi'_A\rangle \\ + \frac{\lambda^2}{2} \sum_K \sum_{ij} \int_0^\infty d\tau\, e^{i\bar{\omega}_{A'K}\tau} Q_{ij}(\tau)\hat{V}_i|\bar{\Phi}_K\rangle\langle\bar{\Phi}_K|\hat{V}_j|\Phi'_A\rangle + \mathcal{O}(\lambda^3), \end{aligned} \tag{S22}$$

where

$$\bar{\omega}_{A'K} = E'_A - \bar{E}_K.$$

Performing the Fourier transform of the response functions gives

$$\begin{aligned} E'_A|\Phi'_A\rangle &= \hat{\bar{H}}_S|\Phi'_A\rangle \\ &\quad - \frac{i\lambda^2}{2}\sum_K\sum_{ij} C_{ij}^{\mathrm{sym}}(\bar{\omega}_{A'K})\hat{V}_i|\bar{\Phi}_K\rangle\langle\bar{\Phi}_K|\hat{V}_j|\Phi'_A\rangle \\ &\quad + \frac{\lambda^2}{2}\sum_K\sum_{ij} Q_{ij}(\bar{\omega}_{A'K})\hat{V}_i|\bar{\Phi}_K\rangle\langle\bar{\Phi}_K|\hat{V}_j|\Phi'_A\rangle + \mathcal{O}(\lambda^3). \end{aligned} \tag{S23}$$

Finally, separating the real and imaginary parts of $Q_{ij}(\omega)$ and using the fluctuation–dissipation relation adopted in the main text, the contribution originating from $C_{ij}^{\mathrm{sym}}(\omega)$ combines with the imaginary part of $Q_{ij}(\omega)$ to produce the frequency-dependent factor $T(\omega) = 1 + \Theta(\omega) - \Theta(-\omega)$, whereas the real part of $Q_{ij}(\omega)$ remains unchanged. We therefore obtain

$$\begin{aligned} E'_A|\Phi'_A\rangle &= \hat{\bar{H}}_S|\Phi'_A\rangle \\ &\quad + \frac{\lambda^2}{2}\sum_K\sum_{ij} \Re\{Q_{ij}(\bar{\omega}_{A'K})\}\,\hat{V}_i|\bar{\Phi}_K\rangle\langle\bar{\Phi}_K|\hat{V}_j|\Phi'_A\rangle \\ &\quad + \frac{i\lambda^2}{2}\sum_K T(\bar{\omega}_{A'K})\sum_{ij} \Im\{Q_{ij}(\bar{\omega}_{A'K})\}\,\hat{V}_i|\bar{\Phi}_K\rangle\langle\bar{\Phi}_K|\hat{V}_j|\Phi'_A\rangle + \mathcal{O}(\lambda^3). \end{aligned} \tag{S24}$$

It is therefore convenient to define the state-specific OQS-PCM(ω) Hamiltonian

$$\begin{aligned} \hat{H}_{\mathrm{OQS}}^{A'} &= \hat{\bar{H}}_S + \frac{\lambda^2}{2}\sum_K\sum_{ij} \Re\{Q_{ij}(\bar{\omega}_{A'K})\}\,\hat{V}_i|\bar{\Phi}_K\rangle\langle\bar{\Phi}_K|\hat{V}_j \\ &\quad + \frac{i\lambda^2}{2}\sum_K T(\bar{\omega}_{A'K})\sum_{ij} \Im\{Q_{ij}(\bar{\omega}_{A'K})\}\,\hat{V}_i|\bar{\Phi}_K\rangle\langle\bar{\Phi}_K|\hat{V}_j, \end{aligned} \tag{S25}$$

which satisfies the nonlinear stationary eigenvalue problem

$$\hat{H}^{A'}_{\mathrm{OQS}}|\Phi'_A\rangle = E'_A|\Phi'_A\rangle + \mathcal{O}(\lambda^3). \tag{S26}$$

The nonlinearity arises from the dependence of $\hat{H}^{A'}_{\mathrm{OQS}}$ on the unknown stationary energy through $\bar{\omega}_{A'K} = E'_A - \bar{E}_K$. Setting $\lambda = 1$ recovers the stationary OQS-PCM(ω) equation presented in the main text.

We emphasize that the stationary OQS-PCM(ω) formulation presented here contains the full polarization contribution but only part of the dispersion interaction, as extensively discussed in Ref. [2]. Specifically, it includes the dispersion contribution arising from the solvent response to solute fluctuations, but not the reciprocal contribution arising from the solute response to solvent fluctuations. The latter term is, however, retained in the full TD-SSE-pol theory. A formal derivation that makes this missing contribution explicit can be obtained by taking the expectation value of the Hamiltonian with respect to the time-dependent solute state, consistently expanding the resulting expression order by order in perturbation theory, and subsequently performing the ensemble average over bath fluctuations. An analogous procedure is carried out by Gaspard and Nagaoka for the reduced density matrix of a general open quantum system.[1]

OQS-PCM(ω) is derived from the equation of motion for the ensemble-averaged coherent amplitude within the complex-noise Gaspard-Nagaoka stochastic formulation. Accordingly, the stochastic forcing does not contribute to this equation through second order in the solute-solvent coupling. Solvent fluctuation effects described at the reduced-density-matrix level, including thermalization, pure dephasing, and the reciprocal dispersion contribution discussed above, lie outside the scope of the present stationary formulation. Furthermore, spectral broadening beyond the dissipation lifetime encoded in $\Im\{E'_A\}$ goes beyond the intended scope of OQS-PCM(ω).

S1.2 Inertial and Dynamic Polarization

Given that $\hat{\hat{H}}_S = \hat{H}_S + \sum_i < \hat{q}_i > \hat{V}_i$, the new system Hamiltonian remains undefined until we specify $< \hat{q}_i >$. To that aim, let us reconsider the solvent operator $\hat{q}_i$. In particular, let us split the solvent degrees of freedom in two components: a *fast* component that adapts to the solute's electronic transitions (also referred to as dynamic, hereafter denoted by the subscript 'dyn'), and a *slow* component that lags behind, remaining in equilibrium with the solute's electronic ground state (also referred to as inertial, hereafter denoted by the subscript 'in') [see Ref. [4] and references therein]. Since the focus of this work is on the electronic excitation of the solute, we can identify the inertial degrees of freedom with those of the solvent nuclei, and the dynamical one with those of solvent electrons. In Ref. [5], nuclei were considered through a classical average. Here in the spirit of Gaspard and Nagaoka approach, we consider instead a typical quantum state of the solvent nuclei (i.e., the inertial degrees of freedom), $|0'_{\text{in}}\rangle$ that represents their polarized state in equilibrium with the ground state of the solute. By definition of inertial degrees of freedom, such state will not change upon solute electronic excitations. Therefore, solvent states can be written as $|0'_{\text{in}}\rangle \otimes |\chi_{\text{dyn}}\rangle$, where $|\chi_{\text{dyn}}\rangle$ is the quantum state of dynamical degrees of freedom (solvent electrons in this work). Considering this factorization and the assumption that only $|0'_{\text{in}}\rangle$ matters, we can write the operator $\hat{q}_i$ as follows:

$$\hat{q}_i = q_{\text{in},i} |0'_{\text{in}}\rangle \langle 0'_{\text{in}}| \otimes \hat{I}_{\text{dyn}} + \hat{I}_{\text{in}} \otimes \hat{q}_{\text{dyn},i} \,. \tag{S27}$$

where $\hat{q}_{\text{dyn},i}$ is an operator acting only on the Hilbert space of the *dynamic states of the solvent*. $\hat{I}_{\text{in}}$ and $\hat{I}_{\text{dyn}}$ are ancillary identity operators acting only on the inertial and dynamic solvent Hilbert spaces, respectively. Note that the inertial contribution is a *constant* term that we write as:

$$q_{\text{in},i} = q_i^{(0)} - q_{\text{dyn},i}^{(0)} \ , \tag{S28}$$

where $q_i^{(0)}$ is the total polarization charge in equilibrium with the ground-state solute, whereas $q_{\mathrm{dyn},i}^{(0)}$ is the *part* of such polarization charge due to the fast degrees of freedom of the solvent. To compute the latter, we rely on the following response equations:

$$q_i^{(0)} = \sum_j Q_{0,ij}\ V_j^0 \ , \tag{S29}$$

$$q_{\mathrm{dyn},i}^{(0)} = \sum_j Q_{d,ij}\ V_j^0 \ , \tag{S30}$$

V_j^0 is the solute's ground-state potential. On the other hand, $Q_{0,ij}$ and $Q_{d,ij}$ are the PCM response matrices that depend on ϵ_0 and ϵ_d, respectively. Here, ϵ_0 denotes the static dielectric constant of the solvent, corresponding to the zero-frequency value of its dielectric function. In contrast, ϵ_d represents the dynamic (high-frequency or optical) dielectric constant, which is often approximated as the average of the dielectric function over the visible range.

With the inertial/dynamic splitting introduced in Eq. (S27), we set

$$\langle \hat{q}_i \rangle = q_{\mathrm{in},i},$$

by choosing the reference solvent state as composed of the inertial polarized state $|0'_{\mathrm{in}}\rangle$ and an unpolarized dynamic component state, i.e., $|0'_{\mathrm{in}}\rangle \otimes |0_{\mathrm{dyn}}\rangle$, with $|0_{\mathrm{dyn}}\rangle$ satisfying

$$\langle 0_{\mathrm{dyn}}|\, \hat{q}_{\mathrm{dyn},i}\, |0_{\mathrm{dyn}}\rangle = 0\,.$$

Dynamic polarization effects are included exclusively in one of the additional terms of the OQS-PCM Hamiltonian of Eq. (11), main text. This choice allows us to write the new system Hamiltonian as

$$\hat{\tilde{H}}_S = \hat{H}_S + \sum_i \hat{V}_i\ q_{\mathrm{in},i}^{(0)}\ . \tag{S31}$$

We call it the inertial-solvent Hamiltonian. Note that for weakly polar solvents (where

$\epsilon_0 \approx \epsilon_d$), $q^{(0)}_{\mathrm{in},i} = 0$, resulting in the gas-phase solute Hamiltonian.

Since the modified system Hamiltonian includes inertial polarization effects, the OQS-PCM corrections must account solely for the dynamic degrees of freedom of the solvent. Therefore, the frequency-dependent dielectric function, appearing in $\Re\{Q(\omega)\}$ and $\Im\{Q(\omega)\}$ of the main text Eq. (11), must be a *filtered* version of the *empirical* dielectric function, retaining only the electronic component of the solvent response. In practice, this filtering is only necessary for polar solvents, where steep Debye-like terms take effect in the low-frequency region of the solvent dielectric function. In such cases, we remove these contributions, as well as any Drude–Lorentz-like components with transition frequencies below the optical range, from the dielectric function used in the calculations.

Since Hamiltonian (S31) represents a modified yet still *closed* system, the energies (namely, $\bar{E}_I$) obtained from its diagonalization are not the correct free energies. To obtain the associated free energies (namely, $\bar{G}_I$), one needs to subtract the work spent by placing the inertial charges on the cavity surface:

$$\bar{G}_I = \bar{E}_I - \frac{1}{2}\sum_i q^{(0)}_{\mathrm{in},i}\; V^0_i \ . \tag{S32}$$

S1.3 Frozen-Solvent Hamiltonian

We have expressed the OQS-PCM Hamiltonian in a basis set composed of the eigenstates of (S31). Such a basis may be obtained by diagonalizing (S31) in the basis of eigenstates of the more familiar frozen-solvent Hamiltonian, i.e.,

$$\hat{H}^f_S = \hat{H}_S + \sum_i \hat{V}_i\; q^{(0)}_i \ . \tag{S33}$$

Using Eq. (S28), we rewrite the Hamiltonian in Eq. (S31) as:

$$\hat{\bar{H}}_S = \hat{H}^f_S - \sum_i \hat{V}_i\; q^{(0)}_{\mathrm{dyn},i} \ . \tag{S34}$$

According to Eq. (S30), the dynamical polarization charges in equilibrium with the solute's ground state read $q^{(0)}_{\text{dyn},i} = \sum_j Q_{d,ij} \left\langle \Phi^f_0 \middle| \hat{V}_j \middle| \Phi^f_0 \right\rangle$, where $\left| \Phi^f_0 \right\rangle$ satisfies $\hat{H}^f_S \left| \Phi^f_0 \right\rangle = E^f_0 \left| \Phi^f_0 \right\rangle$.

We expand the eigenstates of $\hat{\bar{H}}_S$ in the frozen-solvent basis as $\left| \bar{\Phi}_K \right\rangle = \sum_J c^K_J \left| \Phi^f_J \right\rangle$, where $\hat{H}^f_S \left| \Phi^f_J \right\rangle = E^f_J \left| \Phi^f_J \right\rangle$. Inserting this expansion into the time-independent Schrödinger equation for Eq. (S34) leads to the following eigenvalue problem:

$$\bar{E}_I \; c^K_I = \sum_J \bar{H}_{S,IJ} \; c^K_J \; , \tag{S35}$$

with matrix elements:

$$\bar{H}_{S,IJ} = E^f_I \; \delta_{IJ} - \sum_i \left\langle \Phi^f_I \middle| \hat{V}_i \middle| \Phi^f_J \right\rangle \; q^{(0)}_{\text{dyn},i} \; . \tag{S36}$$

Finally, the diagonalization of Eq. (S36) yields the ground and excited states $\left| \bar{\Phi}_K \right\rangle$ and the corresponding energies $\bar{E}_K$ of the Hamiltonian $\hat{\bar{H}}_S$.

S1.4 OQS-Onsager Model

In this section, we obtain the OQS-Onsager formulation by adapting the OQS-PCM expressions to the Onsager model. The Onsager model is still a continuum solvation approach, where the solvent is treated as an infinite material characterized by a dielectric function. However, in this case, the solvent-excluded cavity is spherical, and the solute is approximated as a point dipole.[6] Figure S1 illustrates the difference between PCM and Onsager model. In this case, the interaction Hamiltonian can be rewritten as:

$$\hat{H}_{SB} = - \sum_{i=1}^{3} \hat{\mu}_i \otimes \hat{R}_i \; , \tag{S37}$$

where $\hat{\vec{\mu}}$ is the dipole operator acting on solute states, and $\hat{\vec{R}}$ is an electric field operator acting on solvent states. In particular, the matrix elements of $\hat{\vec{R}}$ represent static and transition electric fields produced by the solvent at the center of the cavity, where the solute dipole is

located. The subindex i in (S37) indicates a Cartesian component of each vectorial operator.

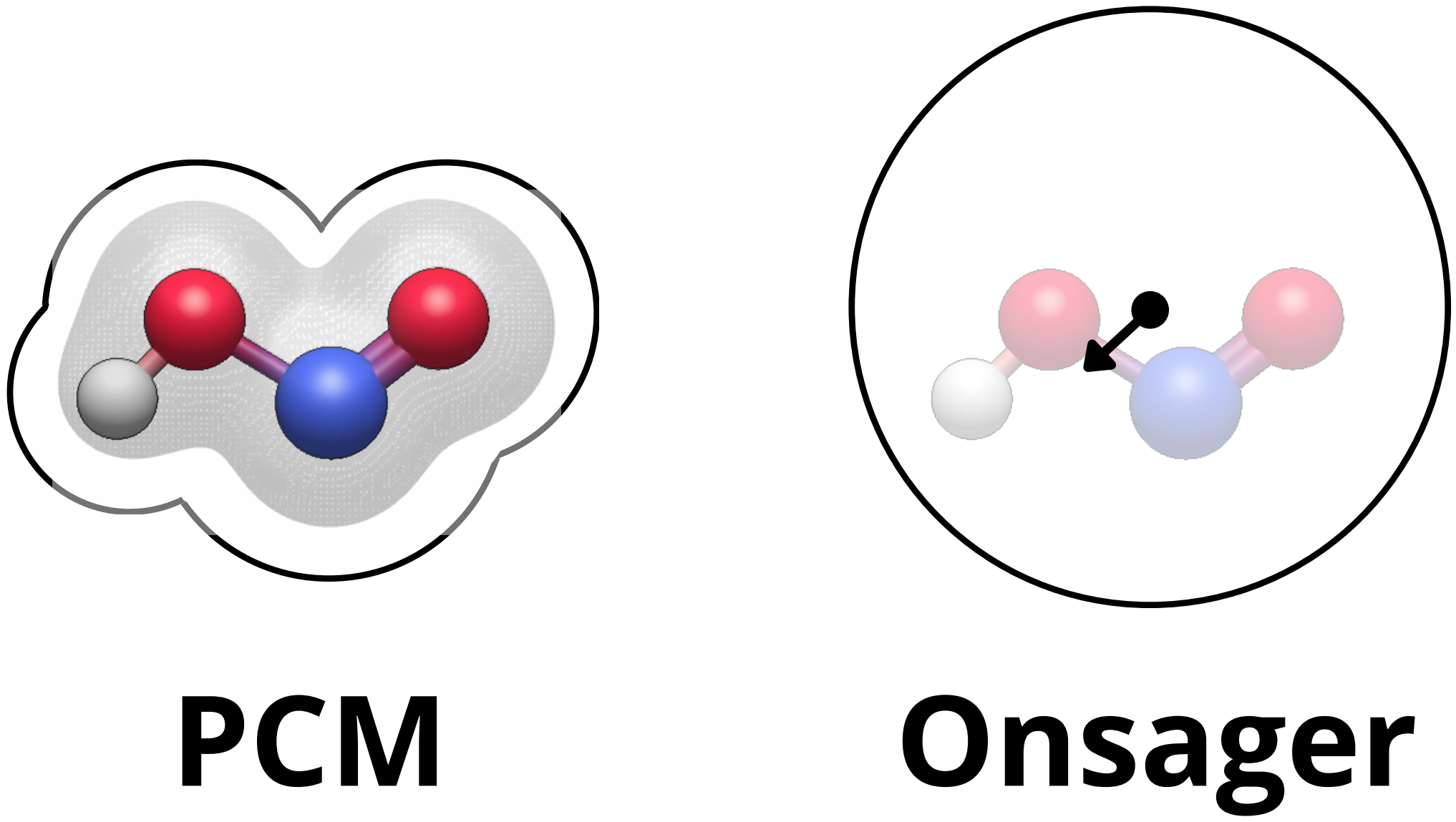

Figure S1: Illustrative comparison of the PCM and Onsager models for nitrous acid (HNO_2). In the PCM depiction, the gray region represents the molecular electronic density, whereas in the Onsager model the arrow at the center of the cavity denotes the molecular dipole moment. The cavity boundary is indicated by a black line, with the surrounding region corresponding to the continuum dielectric solvent. HNO_2 is shown in a standard ball-and-stick representation, with gray, blue, and red spheres denoting hydrogen, nitrogen, and oxygen atoms, respectively. In PCM, the cavity conforms to the molecular shape and accounts for all molecular multipole moments, whereas in the Onsager model the cavity is spherical and only the dipole moment is considered. All geometric features and dipole representations are schematic and intended solely for illustrative purposes.

The comparison of (S37) and main-text's (7) suggests the following mapping to derive the equation of an OQS-Onsager(ω) approach: $\hat{\mu}_i \to \hat{V}_i$ and $-\hat{R}_i \to \hat{q}_i$. In this case, the frequency-dependent PCM response matrix maps onto:

$$f_{ij}(\omega) = -g(\omega)\ \delta_{ij}\ , \tag{S38}$$

where $g(\omega)$ is the Onsager response factor, i.e.,[6]

$$g(\omega) = \frac{2(\epsilon(\omega)-1)}{(2\epsilon(\omega)+1)}\frac{1}{a^3}\ . \tag{S39}$$

This expression depends on the radius of the spherical cavity, a, and the complex-valued frequency-dependent dielectric function, $\epsilon(\omega)$. As commented in Section S1.2, $\epsilon(\omega)$ only takes into account the fast degrees of freedom of the solvent response.

Using the latter correspondences, we arrive at the OQS-Onsager Hamiltonian (with generic polarity) [cf. (13), main text], i.e.,

$$\bar{H}^{A'}_{OQS,IJ} = \bar{E}_I\ \delta_{IJ} - \frac{1}{2}\sum_K \Re\{g(\bar{\omega}_{A'K})\}\left\langle \bar{\Phi}_I\right| \hat{\vec{\mu}} \left|\bar{\Phi}_K\right\rangle \cdot \left\langle \bar{\Phi}_K\right| \hat{\vec{\mu}} \left|\bar{\Phi}_J\right\rangle \ , \\ - \frac{i}{2}\sum_K T(\bar{\omega}_{A'K})\ \Im\{g(\bar{\omega}_{A'K})\}\left\langle \bar{\Phi}_I\right| \hat{\vec{\mu}} \left|\bar{\Phi}_K\right\rangle \cdot \left\langle \bar{\Phi}_K\right| \hat{\vec{\mu}} \left|\bar{\Phi}_J\right\rangle \tag{S40}$$

where $\Re\{g(\omega)\}$ and $\Im\{g(\omega)\}$ are the real and imaginary parts of $g(\omega)$ and $T(\omega)$ is defined in the main text. Moreover, $\bar{E}_K$ and $\left|\bar{\Phi}_K\right\rangle$ are eigenenergies and eigenstates of the inertial-solvent Hamiltonian in the framework of Onsager [cf. (S31)]:

$$\hat{\bar{H}}_S = \hat{H}_S - \hat{\vec{\mu}} \cdot \vec{R}^{(0)}_{\text{in}} \ , \tag{S41}$$

where $\vec{R}^{(0)}_{\text{in}}$ is the inertial reaction field in equilibrium with the solute's ground-state, i.e., $\vec{R}^{(0)}_{\text{in}} = (g_0 - g_d)\ \vec{\mu}_0$ with $\vec{\mu}_0 = \left\langle \Phi^f_0 \right| \hat{\vec{\mu}} \left| \Phi^f_0 \right\rangle$. Here, $g_0 = 2(\epsilon_0 - 1)/(2\epsilon_0 + 1)/a^3$ and $g_d = 2(\epsilon_d - 1)/(2\epsilon_d + 1)/a^3$. On the other hand, $\left|\Phi^f_0\right\rangle$ satisfies $(\hat{H}_S - \hat{\vec{\mu}} \cdot \vec{R}_0)\left|\Phi^f_0\right\rangle = E^f_0 \left|\Phi^f_0\right\rangle$ with $\vec{R}_0 = g_0\ \vec{\mu}_0$.

We will also consider (S40) under the BO-sol and SS-pol approximations, given in main-text Eqs. (16)-(17), in which the role of the dynamical PCM response function is played by g_d (see section 2.1 in the main text). Hereafter, we will use the term OQS-Continuum to refer collectively to both the OQS-PCM and OQS-Onsager approaches.

S2 Additional Computational Details

S2.1 Frozen-Solvent Calculations: Onsager Model

We solve the frozen-solvent Hamiltonian eigenproblem for the Onsager model (S41) using Linear-Response Time-Dependent Density Functional Theory (LR-TDDFT) at the LC-ξBPBE/6-31+G(d,p) level, within a locally modified version of GAMESS-US.[7] As in the case of PCM, we request the full space of singlet excited states. (See the *Computational details* section in the main text for PCM.) The diameter of Onsager's cavity is determined for each molecule from a volume calculation in Gaussian 16. Since GAMESS-US implements Onsager only for ground states, we first perform a self-consistent DFT calculation to extract the ground-state dipole, $\vec{\mu}_0$. We then run a DFT and LR-TDDFT calculation in vacuo subject to an external electric field $\vec{R}_0$, set equal to the solvent reaction field in equilibrium with the ground-state solute according to the Onsager model, i.e., $\vec{R}_0 = g_0\,\vec{\mu}_0$. This procedure effectively simulates the frozen-solvent approximation for the Onsager model.

S2.2 Post-Processing of PCM and Onsager Frozen-Solvent Results: Transition Dipoles and Potentials

After solving the frozen-solvent eigenproblem, we collect the excitation energies, $E_K^f - E_0^f$, along with the static and transition electric dipole moments, $\langle\Phi_I^f|\hat{\vec{\mu}}|\Phi_J^f\rangle$, and electrostatic potentials, $\langle\Phi_I^f|\hat{V}_i|\Phi_J^f\rangle$. For transitions where neither I nor J corresponds to the ground state, the Slater-Condon rules are applied (see, e.g., Ref. [8]), i.e., orbital relaxation upon excitation is neglected. The modified GAMESS-US implementation (see the *Computational details* section in the main text) allows extraction of potentials for all state combinations, including excited-to-excited transitions, at each PCM cavity tessera. Excited and excited-to-excited dipoles are computed in an external code that processes atomic-orbital (AO) integrals, molecular orbital expansions in AOs, and LR-TDDFT excitation and de-excitation amplitudes. These quantities are then used to construct the inertial-solvent Hamiltonian and

also contribute to the calculation of electronic absorption spectra.

S2.3 From Frozen-Solvent to the Inertial-Solvent Basis Set

As outlined in Sec. S1.3, the inertial-solvent states, $\left|\bar{\Phi}_K\right\rangle$, i.e., the eigenstates of the inertial-solvent Hamiltonian (S31), are obtained as linear combinations of frozen-solvent states $\left|\bar{\Phi}_I\right\rangle = \sum_J c_J^I \left|\Phi_J^f\right\rangle$. From the frozen-solvent ground state, we compute the potentials and dipoles, $V_j^0 = \left\langle \Phi_0^f \right| \hat{V}_j \left|\Phi_0^f\right\rangle$ and $\vec{\mu}_0 = \left\langle \Phi_0^f \right| \hat{\vec{\mu}} \left|\Phi_0^f\right\rangle$. The Hamiltonian $\hat{\bar{H}}_S$ [Eqs. (S31) and (S34)] is then fully specified using $q_{\mathrm{in},i}^{(0)} = \sum_j (Q_{0,ij} - Q_{d,ij}) V_j^0$ and $\vec{R}_{\mathrm{in}}^{(0)} = (g_0 - g_d)\vec{\mu}_0$ for the PCM and Onsager models, respectively. Diagonalization of $\hat{\bar{H}}_S$ yields the excited-state energies $\bar{E}_I$ and the corresponding states $\left|\bar{\Phi}_I\right\rangle$, expressed in the frozen-solvent basis $\left\{\left|\Phi_K^f\right\rangle\right\}$. Finally, the static and transition potentials and dipoles in the inertial-solvent basis, entering the OQS-PCM and OQS-Onsager Hamiltonians [main text Eq. (13) and (S40)], are expanded in terms of their frozen-solvent counterparts.

S2.4 Frequency-Dependent Dielectric Functions of solvents

For the full-frequency OQS-Continuum approach, we require an analytical expression approximating the frequency dependence of the dielectric function, $\epsilon(\omega)$, for the studied solvents. Recall that $\epsilon(\omega)$ should reflect only the fast component of the solvent polarization response. In the case of water, a fit including 2 Debye and 19 Drude-Lorentz terms for the dielectric function, spanning a host of experimental data in the range 0-100 eV, was published recently.[9] We neglect Debye's and the IR-range Drude-Lorentz terms, as these correspond to slow degrees of freedom of the solvent. In the case of benzene, we fit a single experimental dataset taken from Ref. [10] (in the range 1-100 eV) with 6 Drude-Lorentz terms. The fit is performed using only the real part of the dielectric function data, yet it accurately captures the behavior of the imaginary part as well. Since all Drude-Lorentz terms for benzene are associated with fast components of the solvent polarization, we keep them all. Static and dynamic dielectric constants are then estimated from the zero-frequency limit and the

average real part over the optical range of $\epsilon(\omega)$, respectively.

S2.5 OQS-Continuum(ω) Algorithm

As stated in the main text, the OQS-PCM(ω) Hamiltonian is state-specific and depends nonlinearly on the energy of the target solvated state, as does the corresponding Onsager model. To solve this problem one must diagonalize a different Hamiltonian for each solvated state, and pick the corresponding solvated state out from the resulting spectrum. The nonlinear nature of the Hamiltonian requires solving the problem iteratively, starting from the inertial-solvent energy of the desired state. Here, we provide the algorithmic OQS-Continuum(ω) workflow explicitly:

1. Set $n = 1$, and take the A-th inertial-solvent energy as the first approximation of the solvated-state energy, namely $E_A'^{(n)} = \bar{E}_A$.

2. Compute the OQS-Continuum Hamiltonian for the A-th solvated state in the current iteration. For OQS-PCM,

$$\begin{aligned} H_{IJ}\left(E_A'^{(n)}\right) &= \bar{E}_I\ \delta_{IJ} \\ &+ \frac{1}{2}\sum_K \sum_{i,j} \Re\left\{Q_{ij}\left(E_A'^{(n)} - \bar{E}_K\right)\right\} \langle\bar{\Phi}_I|\hat{V}_i|\bar{\Phi}_K\rangle\langle\bar{\Phi}_K|\hat{V}_j|\bar{\Phi}_J\rangle \\ &+ \frac{i}{2}\sum_K T\left(E_A'^{(n)} - \bar{E}_K\right)\sum_{i,j} \Im\left\{Q_{ij}\left(E_A'^{(n)} - \bar{E}_K\right)\right\} \langle\bar{\Phi}_I|\hat{V}_i|\bar{\Phi}_K\rangle\langle\bar{\Phi}_K|\hat{V}_j|\bar{\Phi}_J\rangle\ , \end{aligned} \tag{S42}$$

   For OQS-Onsager, one starts from (S40) and performs analogous substitutions.

3. Diagonalize (S42), obtaining the eigenvalues λ and eigenvectors $\vec{v}$ that satisfy

$$\lambda\ v_I = \sum_J H_{IJ}\left(E_A'^{(n)}\right)\ v_J\ . \tag{S43}$$

4. Project the solvated states I' onto the initial inertial-solvent reference, $\langle\bar{\Phi}_A|\Phi'_I\rangle = v_A^{I'}$. Identify the state A' as the I' that maximizes $|v_A^{I'}|^2$, and set $E_A^{\prime(n+1)} = \lambda_{I'}$.

5. Set $n = n + 1$ and loop from 2 to 4 until convergence is reached; specifically,

$$\left|\Re\left\{E_A^{\prime(n+1)}\right\} - \Re\left\{E_A^{\prime(n)}\right\}\right| < \text{tolerance}\ , \tag{S44}$$

where $\Re\left\{E_A^{\prime(n)}\right\}$ denotes the real part of $E_A^{\prime(n)}$. In this work, the tolerance is set to 10^{-6} Hartree.

6. Once (S44) is satisfied, set the converged solvated energy to $E'_A = E_A^{\prime(n+1)}$. The associated eigenvector from the last iteration provides the expansion coefficients of $|\Phi'_A\rangle$ in the inertial-solvent basis, $C_I^{A'} = v_I^{A'}$, with

$$|\Phi'_A\rangle = \sum_I C_I^{A'} \left|\bar{\Phi}_I\right\rangle\ . \tag{S45}$$

As such, the latter algorithm suffers from slow or even failed convergence. In practice, such issues are cured by mixing the results of previous iterations to stabilize the current one. Taking into account our specific mixing implementation, steps 2-3 are amended as follows:

2′. Compute

$$\begin{aligned} H_{IJ}(\varepsilon) &= \bar{E}_I\ \delta_{IJ} \\ &+ \frac{1}{2}\sum_K \sum_{i,j} \Re\left\{Q_{ij}\left(\varepsilon - \bar{E}_K\right)\right\} \left\langle\bar{\Phi}_I\right| \hat{V}_i \left|\bar{\Phi}_K\right\rangle \left\langle\bar{\Phi}_K\right| \hat{V}_j \left|\bar{\Phi}_J\right\rangle \\ &+ \frac{i}{2}\sum_K T\left(\varepsilon - \bar{E}_K\right) \sum_{i,j} \Im\left\{Q_{ij}\left(\varepsilon - \bar{E}_K\right)\right\} \left\langle\bar{\Phi}_I\right| \hat{V}_i \left|\bar{\Phi}_K\right\rangle \left\langle\bar{\Phi}_K\right| \hat{V}_j \left|\bar{\Phi}_J\right\rangle\ , \end{aligned} \tag{S46}$$

with

$$\varepsilon = \begin{cases} E_A'^{(n)}, & \text{for } n \leq m \ , \\ \frac{1}{m} \sum_{i=n-m+1}^{n} E_A'^{(i)}, & \text{otherwise} \ . \end{cases}$$

In practice, we mix the last 10 iterations ($m = 10$).

3$'$. Diagonalize $\lambda \ v_I = \sum_J H_{IJ} \left(\xi\right) \ v_J$.

To get the solvated spectrum, we need to run the above algorithm for every state. Since the expansion coefficients of each solvated state in the inertial-solvent basis results from diagonalizing different (state-specific) Hamiltonians, the solvated states are in general nonorthogonal, i.e., $\langle \Phi_I' | \Phi_J' \rangle \neq \delta_{IJ}$. In the case of BO-sol, which does not yield a state-specific eigenproblem, nonorthogonal solvated states can only arise due to degeneracies in the spectrum.

The OQS-Continuum eigenproblem in Eq. (S43) is complex symmetric, owing to the real and symmetric matrix elements of the electrostatic potential and electric dipole moment operators. The appropriate normalization condition for this problem is $\vec{C}^{I'} \cdot \vec{C}^{I'} = 1$, where no Hermitian conjugation appears. As already pointed out in the main text, this means that determining the right eigenvectors, denoted here as $\vec{C}^{I'}$, is sufficient for the calculation of any derived property (see the next section for the case of the oscillator strength).

To solve the OQS-Continuum eigenproblem within the full-frequency, cutoff, BO-sol, and SS-pol approaches, and under both Onsager and PCM frameworks, we implemented an original code[11] in Wolfram Mathematica[12] (v. 11).

S2.6 Electronic and Vibronic Corrections in OQS-PCM Absorption Spectrum Calculations

The electronic oscillator strength for typically Hermitian electronic structure methods is computed as

$$f_A = \frac{2}{3}\,\omega_{0A}\,|\vec{\mu}_{0A}|^2\ , \tag{S47}$$

where ω_{0A} and $\vec{\mu}_{0A}$ are the electronic transition energy and electric dipole moment for the solvated state A. The transition dipole is evaluated as

$$\vec{\mu}_{0A} = \sum_{I,J} C_I^{0'*} C_J^{A'} \left\langle \bar{\Phi}_I \right| \hat{\vec{\mu}} \left| \bar{\Phi}_J \right\rangle\ . \tag{S48}$$

For the non-Hermitian, complex-symmetric OQS-PCM(ω) Hamiltonian, the corresponding oscillator strength is instead computed from

$$f_A = \frac{2}{3}\,\Re\left\{(E'_A - E'_0)\,\vec{\mu}_{0A}\cdot\vec{\mu}_{0A}\right\}\ , \tag{S49}$$

where $\vec{\mu}_{0A}$ is evaluated from the c-product-normalized right eigenvectors as

$$\vec{\mu}_{0A} = \sum_{I,J} C_I^{0'} C_J^{A'} \left\langle \bar{\Phi}_I \right| \hat{\vec{\mu}} \left| \bar{\Phi}_J \right\rangle\ . \tag{S50}$$

Notice that no complex conjugation is introduced in Eqs. (S49) and (S50).

The resulting electronic absorption spectrum for OQS-PCM(ω), cutoff, BO-sol or SS-pol approaches is then obtained by convoluting Gaussian functions centered on ω_{0A} and with intensity f_A for all states A.

For small molecules like those in our test set, nuclear motion can significantly affect absorption energies and intensities; therefore, vibronic effects are included when comparing with experimental spectra. The vibronic treatment described below is entirely based on Hermitian electronic-structure calculations, with ground- and excited-state solvation described by standard and linear-response PCM, respectively. OQS-PCM(ω) and its derived approximations are applied *a posteriori* to introduce further solvation corrections to the transition energy of the main vibronic band.

In the vibronic treatment, the effective transition dipole under the Franck–Condon (FC)

approximation is

$$\vec{\mu}^{vib.}_{0u,Av}(Q) = \vec{\mu}_{0A}(Q) \times \langle 0u|Av\rangle \ , \tag{S51}$$

where $0u$ and Av denote vibronic state u of the electronic ground state and vibronic state v of the A electronic excited state. The FC factors $\langle 0u|Av\rangle$ are the vibrational overlaps between the two states. We explicitly emphasize the parametric dependence of both the electronic and vibronic transition dipoles on the nuclear geometry Q. In many cases the electronic transition dipole $\vec{\mu}_{0A}(Q)$ depends only weakly on Q, but this dependence becomes essential when dealing with electronically dark states, for which $|\vec{\mu}_{0A}(Q)| = 0$ at the ground-state equilibrium geometry. In such cases, intensity is gained through the Herzberg–Teller (HT) contribution, obtained by expanding the transition dipole to first order in Q.

Vibronic effects also shift transition energies, which take the form $\omega_{0uAv}(Q) = E_A(Q) + \varepsilon_{Av} - E_0(Q) - \varepsilon_{0u}$, where $E_A(Q)$ and $E_0(Q)$ are the electronic energies at geometry Q, whereas ε_{Au} and ε_{0u} are the energies of the corresponding vibronic states Av and $0u$ relative to their respective electronic Potential Energy Surface (PES) minima. A common approach is to approximate the ground- and excited-state PES harmonically around their respective minima (the adiabatic Hessian, AH, model). However, because absorption is a vertical process, $\omega_{0uAv}(Q)$ must ultimately be evaluated at the ground-state equilibrium geometry Q_0. This requires extrapolating the excited-state PES, which is represented by its Taylor expansion around the excited-state geometry Q_A, to Q_0. When Q_A and Q_0 are significantly displaced, this extrapolation becomes inaccurate, and a purely vertical description of the excited-state PES, built from the energy, gradient, and Hessian at Q_0 (the vertical Hessian, VH, model), typically yields more reliable spectra. For consistency, the electronic transition dipoles $\vec{\mu}_{0A}(Q)$, assumed to vary smoothly with Q, are evaluated at $Q = Q_0$ and $Q = Q_A$ for the VH and AH model, respectively. See Refs. [13,14] for additional methodological details involved in the vibronic structure calculation.

The S0-S1 transition of the HCl molecule is an example where the AH model fails; we

therefore use the VH. The S0-S1 transition of CH_2O, on the other hand, is electronically dark but vibrationally allowed, hence requiring the HT approximation. In summary, we use the AH model under the FC approximation for HONO, the AH model under the HT approximation for CH_2O and the VH under the FC approximation for HCl.

To compute vibronic effects, we proceed as follows. We perform geometry optimizations and vibrational frequency calculations in Gaussian 16 for both the ground state (S_0) and the first excited state (S_1). In addition, we compute the S_1 energy, gradient and Hessian at the S_0 equilibrium geometry (obtained in practice via a Gaussian 'freq' calculation). All of these calculations include standard PCM solvation effects. The corresponding checkpoint files provide all the relevant ingredients required –electronic energies, gradients, Hessians and transition dipole moments– to compute the vibronic absorption spectrum using the FCClasses code.[14,15]

To construct the excited-state PES for vibronic calculations, we combine information from two electronic structure methods. MP2/6-31+G(d,p) provides the reference geometry and harmonic description for the ground-state PES. Excited-state transition energies, gradients, and Hessians are obtained at the LC-ξBPBE/6-31+G(d,p) level of theory. Our strategy to represent the excited-state PES is as follows:

$$E_1(Q) = E_0^{\text{MP2}}(Q_0) + \left(E_1^{\text{TDDFT}}(Q_0) - E_0^{\text{DFT}}(Q_0)\right) + \left(E_1^{\text{TDDFT}}(Q) - E_1^{\text{TDDFT}}(Q_0)\right) , \tag{S52}$$

where the first term is the MP2 ground-state energy, the second term is the TDDFT vertical excitation energy at the ground-state geometry Q_0, and $E_1^{\text{TDDFT}}(Q)$ is the TDDFT excited-state energy at an arbitrary geometry Q. Evaluated at the ground-state equilibrium geometry Q_0, this expression reduces to

$$E_1(Q_0) = E_0^{\text{MP2}}(Q_0) + (E_1^{\text{TDDFT}}(Q_0) - E_0^{\text{DFT}}(Q_0)) , \tag{S53}$$

which gives the reference MP2 energy shifted by the TDDFT vertical excitation energy. At the excited-state equilibrium geometry Q_1, Eq. (S52) becomes

$$E_1(Q_1) = E_0^{\mathrm{MP2}}(Q_0) + \left(E_1^{\mathrm{TDDFT}}(Q_1) - E_0^{\mathrm{DFT}}(Q_0)\right) \ , \tag{S54}$$

where the difference $\left(E_1^{\mathrm{TDDFT}}(Q_1) - E_0^{\mathrm{DFT}}(Q_0)\right)$ corresponds to the TDDFT adiabatic excitation energy. We note that, at this geometry $Q = Q_1$, the third term of the general expression corresponds to the TDDFT reorganization (relaxation) energy.

MP2 and DFT ground-state geometries yield different low-lying TDDFT excitation energies. After accounting for both solvatochromic and vibrational effects, MP2-based results generally show better agreement with experiment. Because our primary goal is to benchmark realistic solvatochromically shifted bands stemming from our OQS-PCM(ω) approaches against experimental data, this MP2/DFT hybrid methodology for computing vibronic effects provides an appropriate model.

Having computed the vibronic spectrum at the level of standard PCM, we incorporate the solvation effects from the OQS-PCM approach into the transition energy of the main vibronic band as follows:

$$\omega_{0uAv}^{\mathrm{OQS-PCM}}(Q_0) = \omega_{0uAv}(Q_0) - \omega_{0A}^{\mathrm{TDDFT/PCM}}(Q_0) + \omega_{0A}^{\mathrm{OQS-PCM}}(Q_0) \ , \tag{S55}$$

where $\omega_{0A}^{\mathrm{TDDFT/PCM}}(Q_0)$ is the vertical excitation energy obtained from a TDDFT calculation with standard PCM in Gaussian 16 at the ground state equilibrium geometry, and $\omega_{0A}^{\mathrm{OQS-PCM}}(Q_0)$ is the corresponding vertical excitation energy from the OQS-PCM calculation. The rule in (S55) is analogously applied for SS-pol and BO-sol approximations.

S2.7 Full Dispersion Effects in OQS-PCM

In OQS-PCM(ω), the solute-solvent dispersion included arises from the solvent response to solute fluctuations. The complementary contribution, stemming from the solute response

to solvent fluctuations can be approximated effectively by the LR-PCM correction,[2,5,16] as done in the cLR^2 model.[17] When comparing with experimental data, we also include this contribution as a correction to $\omega_{0u1v}^{\mathrm{OQS-PCM}}(Q_0)$ in Eq. (S55). SS-pol is never corrected by this term, preserving its interpretation as a polarization-only approach, and in the case of BO-sol this contribution is not included either, since under its validity conditions it is strictly zero. For all the systems studied, however, this additional term is almost negligible compared to the dispersion shift already provided by OQS-PCM(ω).

S3 Additional Results

S3.1 PCM vs. Onsager Model

We now consider a comparison between two implicit solvation models: the more general PCM and the approximate Onsager model (see Sec. S1.4). Figure S2 shows the UV absorption spectrum of nitrous acid in water computed with the SS-pol, BO-sol and full-frequency OQS-Onsager models. As is apparent, the SS-pol and full-frequency OQS spectra obtained with the Onsager model do not differ markedly from their PCM counterparts [cf. main text Fig. 5, top left]. However, we observe that BO-sol generally produces lower excitation energies than in the case of PCM –albeit systematically overestimated with respect to OQS-Onsager(ω). Moreover, both PCM and Onsager show an average blue shift in BO-sol with respect to SS-pol, but the shift is substantially reduced for Onsager (from 1.15 eV for PCM to 0.24 eV for Onsager). This indicates that nonequilibrium descriptions of the medium interact in a non-trivial way with the cavity shape, as also noted in Ref. [18].

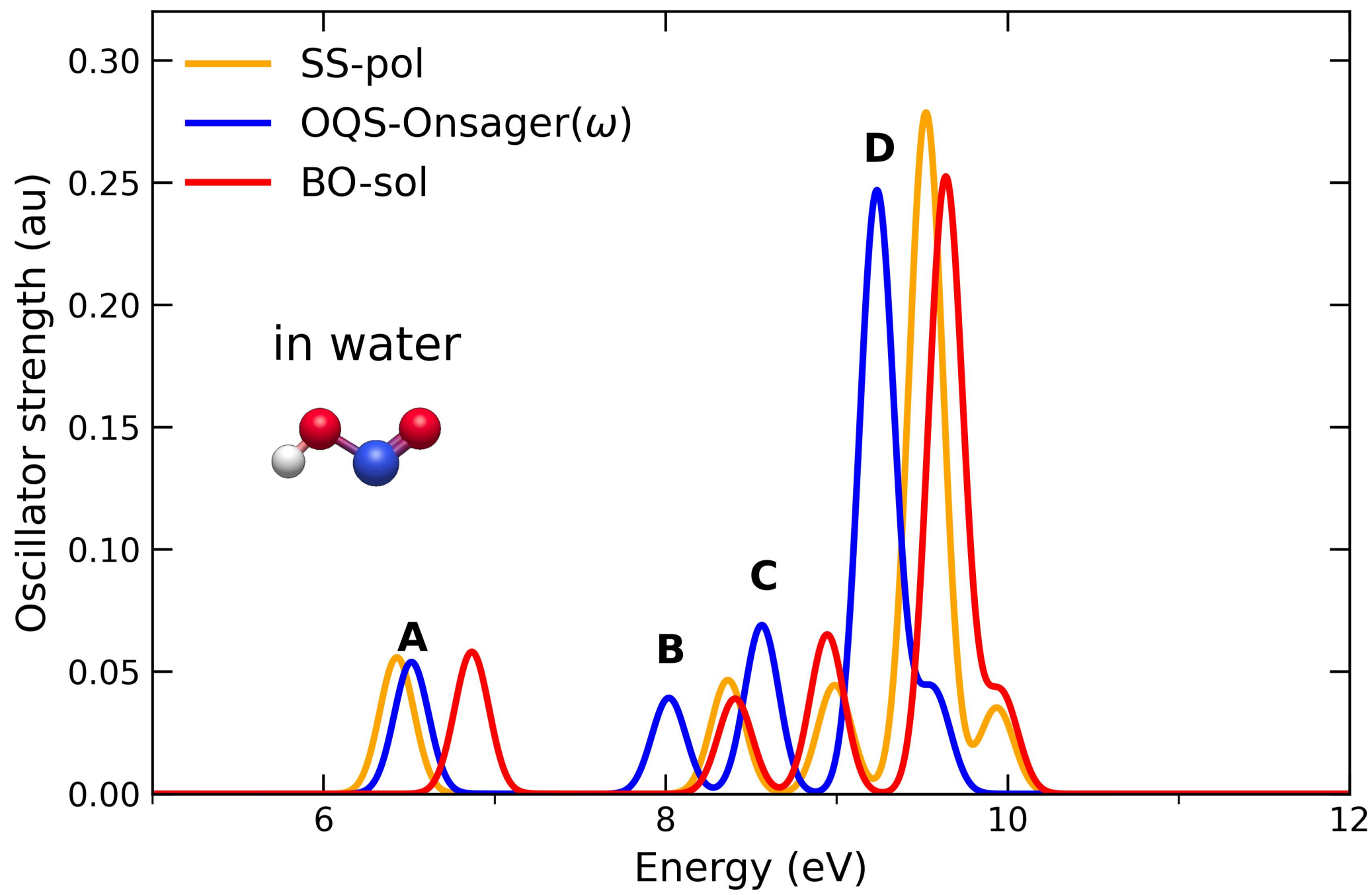

Figure S2: UV absorption spectrum of nitrous acid in water stemming from OQS-Onsager(ω) and its limiting regimes, SS-pol and BO-sol. See also the caption of Fig. 5 left in the main text.

S3.2 Formaldehyde and Hydrochloric Acid in Water and Benzene

In the following, we show results analogous of the main text figures Fig. 4 for formaldehyde and hydrochloric acid molecules.

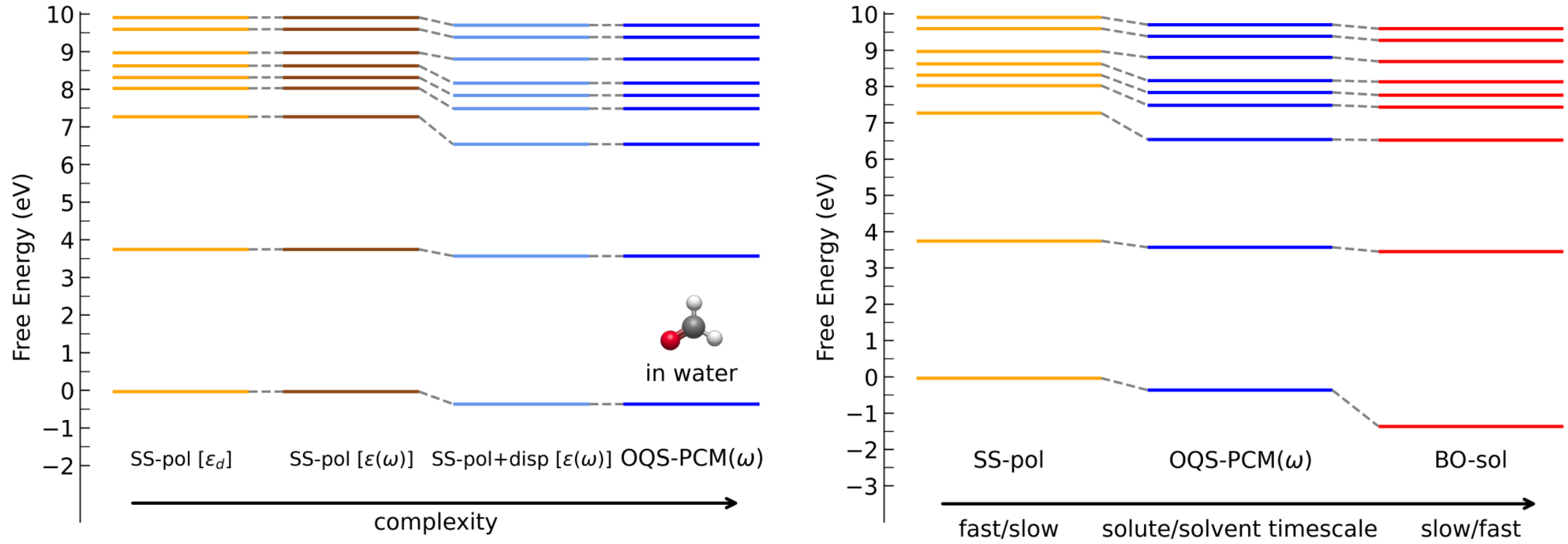

Figure S3: Low-lying singlet free-energy spectrum of formaldehyde in water. Left panel shows results for 'SS-pol [ϵ_d]', 'SS-pol [$\epsilon(\omega)$]', 'SS-pol+disp [$\epsilon(\omega)$]' and OQS-PCM(ω) approaches. Models are ordered along the x-axis in order of increasing complexity. Right panel replots results for SS-pol (same as 'SS-pol [ϵ_d]') and OQS-PCM(ω), and additionally includes BO-sol results. Conceptually, the x-axis spans the solute/solvent timescales, ranging from the fast/slow (SS-pol) to the slow/fast (BO-sol) regimes. Hamiltonians were diagonalized using 50 states with a resolution of the identity spanning all singlet states under the 6-31+G(d,p) basis set, namely $N = 321$.

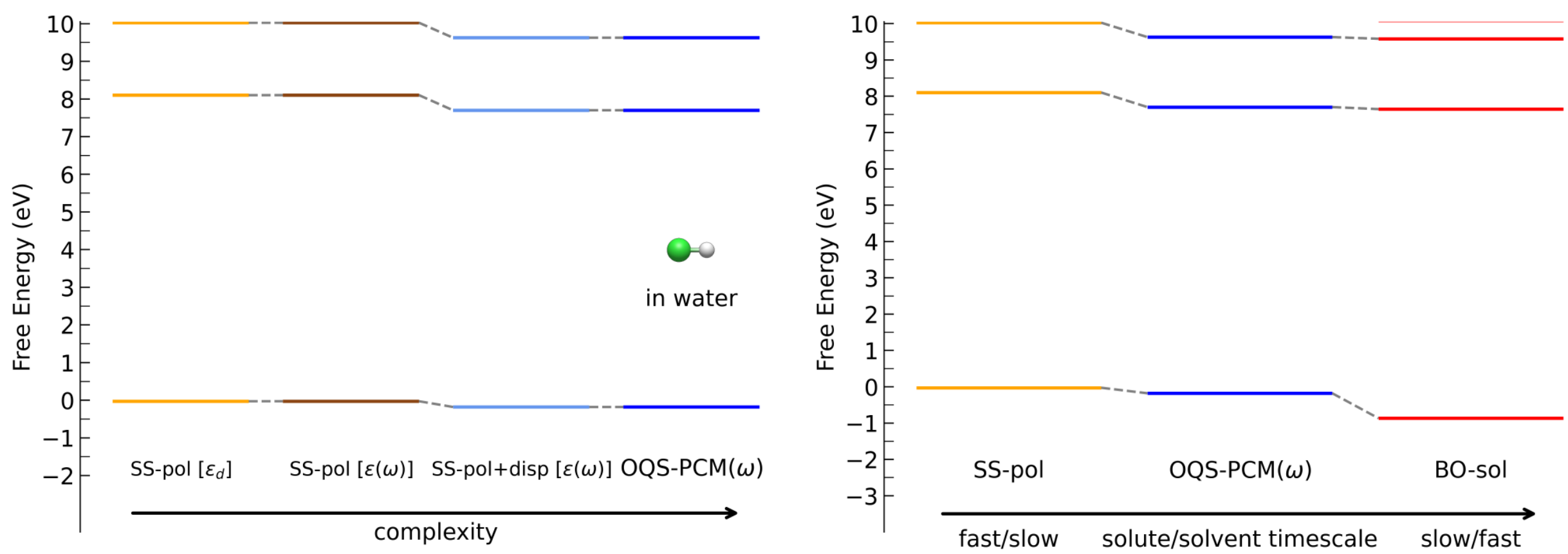

Figure S4: Low-lying singlet free-energy spectrum of hydrochloric acid in water. Left panel shows results for 'SS-pol [ϵ_d]', 'SS-pol [$\epsilon(\omega)$]', 'SS-pol+disp [$\epsilon(\omega)$]' and OQS-PCM(ω) approaches. Models are ordered along the x-axis in order of increasing complexity. Right panel replots results for SS-pol (same as 'SS-pol [ϵ_d]') and OQS-PCM(ω), and additionally includes BO-sol results. Conceptually, the x-axis spans the solute/solvent timescales, ranging from the fast/slow (SS-pol) to the slow/fast (BO-sol) regimes. Hamiltonians were diagonalized using 50 states with a resolution of the identity spanning all singlet states under the 6-31+G(d,p) basis set, namely $N = 172$.

**System**	**Frozen sol.**	**LR**	**cLR**	**SS-pol**	**BO-sol**	**OQS-PCM(ω)**
HNO_2 in vacuo	–	3.27	–	–	–	–
HNO_2 in water	3.31	3.30	3.31	3.29	4.76	3.43
HNO_2 in benzene	3.28	3.27	3.28	3.26	5.18	3.27
CH_2O in vacuo	–	3.70	–	–	–	–
CH_2O in water	3.83	3.83	3.83	3.78	4.82	3.93
CH_2O in benzene	3.75	3.75	3.75	3.68	5.04	3.66
HCl in vacuo	–	7.91	–	–	–	–
HCl in water	8.28	8.26	8.28	8.13	8.51	7.88
HCl in benzene	8.08	8.06	8.08	7.84	8.40	7.47

Table S1: $S_0 \rightarrow S_1$ excitation energies (eV) computed with the frozen-solvent, LR, cLR, SS-pol, BO-sol, and OQS-PCM(ω) approaches. LR values in vacuo are included as reference. LR, cLR, and frozen-solvent results were obtained at the LR-TDDFT level using the LC-ξPBE functional and the 6-31+G(d,p) basis set in GAMESS-US. The SS-pol, BO-sol, and OQS-PCM(ω) methods use the singly excited singlet manifold from the frozen-solvent calculation as their electronic basis (see Computational Details for further discussion).

S3.3 Comparison of LR, cLR, SS-pol, BO-sol and OQS-PCM(ω)

In this section, we compare the excitation energies obtained from the frozen-solvent approximation,[16,19] LR-PCM[20] and cLR-PCM,[5,16,19] as well as SS-pol, BO-sol, and OQS-PCM(ω). For each molecule–solvent combination, the first singlet excitation ($S_0 \rightarrow S_1$) is reported in Table S1.

We note that SS-pol closely matches cLR-PCM, as expected from the fact that cLR accounts for state-specific polarization corrections to the frozen-solvent limit (deviations are around 0.095 eV on average). In contrast, LR-PCM includes an approximate dispersion-like correction arising from the solute response to solvent fluctuations.[2,17] These LR dispersion corrections are small (on the order of $\sim$0.01 eV), and thus generally negligible compared to the state-specific dispersion captured by OQS-PCM(ω), originating from the solvent response to solute fluctuations. HNO_2 represents an exception, where linear-response dispersion contribution is slightly larger than the state-specific one and opposite in sign.

As discussed in the main text, the BO-sol approach systematically overestimates excitation energies, reflecting its use of a frequency-averaged dielectric response that misrepresents dispersion effects. OQS-PCM(ω), by contrast, includes full-frequency polarization, dispersion, and dissipation, and therefore provides the most accurate and physically balanced results among the models compared.

S3.4 Convergence with the Number of Excited States: Other Systems

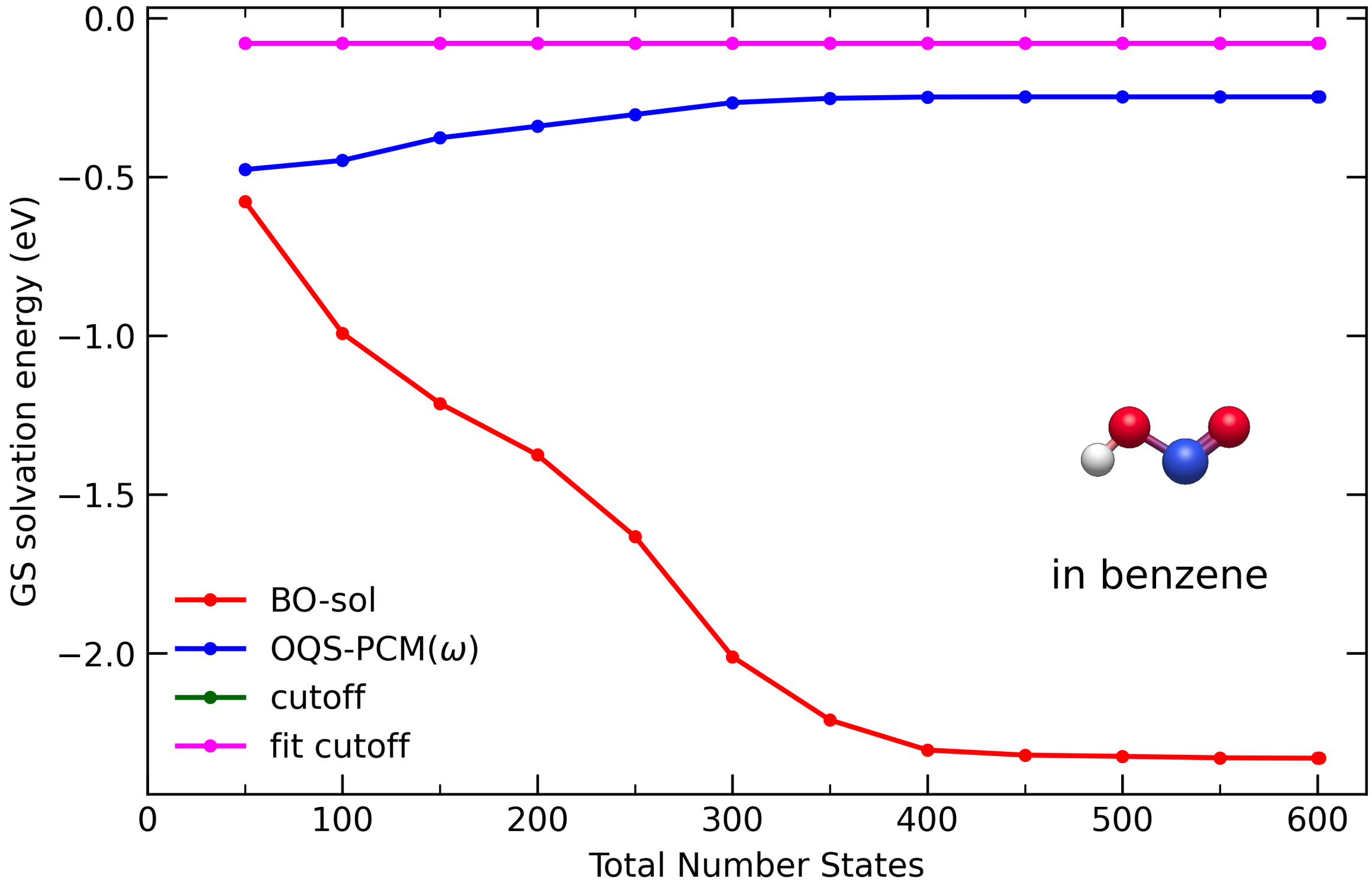

Figure S5: GS solvation free energy as a function of the total number of excited states included in the resolution of the identity for formaldehyde in benzene. Results are shown for OQS-PCM(ω), BO-sol, and the cutoff approaches. On plotted scale, the cutoff (dark green) and fitted cutoff (magenta) results are superimposed. See also caption of Fig. 8 (left) in the main text for nitrous acid in water.

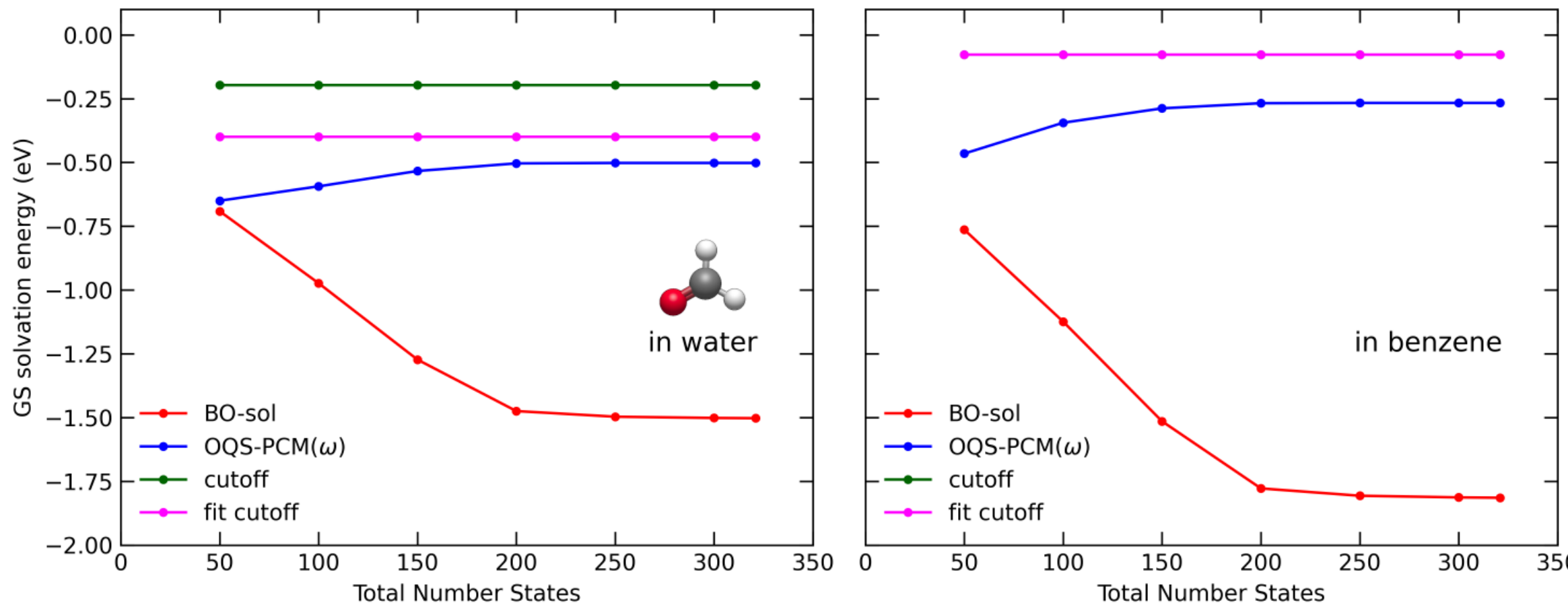

Figure S6: GS solvation free energy as a function of the total number of excited states included in the resolution of the identity for formaldehyde in water (left) and benzene (right). Results are shown for OQS-PCM(ω), BO-sol, and the cutoff approaches. The x axis is sampled in increments of 50 states and includes the point corresponding to the full space of singly excited singlet states (321 states for CH_2O with the 6-31+G(d,p) basis set). On the plotted scale, the cutoff (dark green) and fitted cutoff (magenta) results for benzene are superimposed.

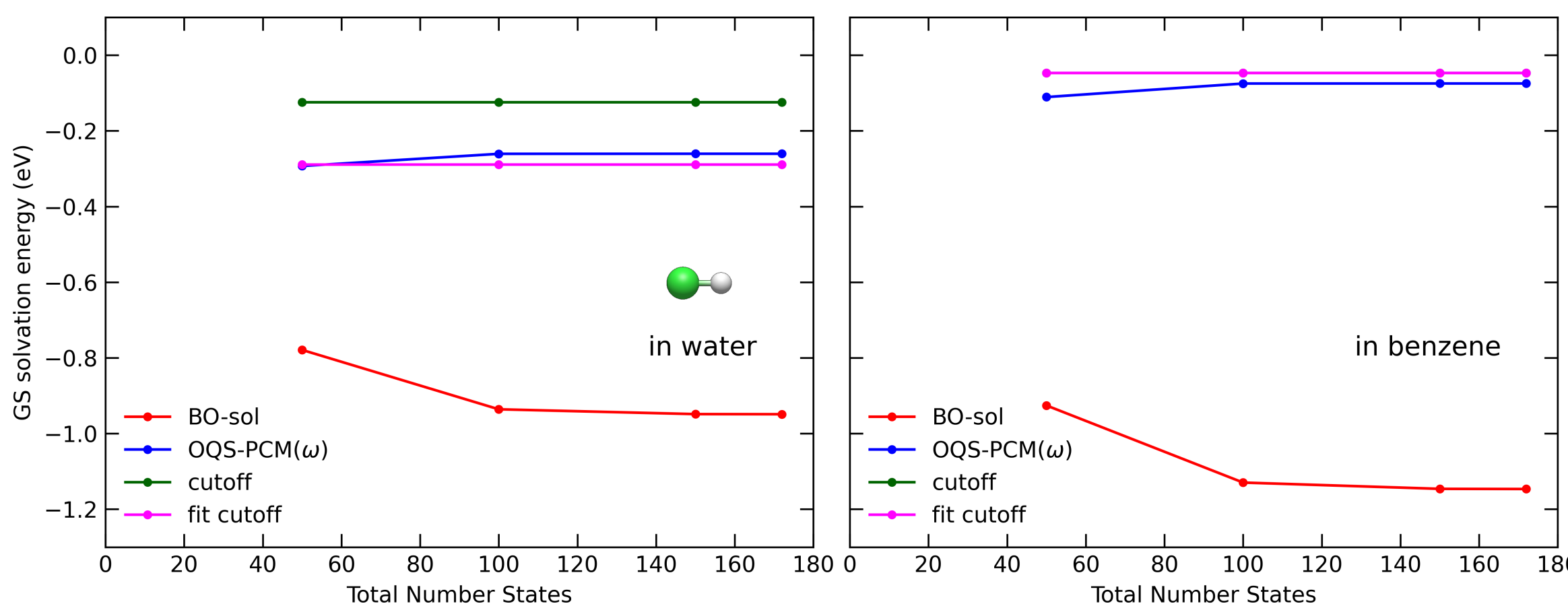

Figure S7: GS solvation free energy as a function of the total number of excited states included in the resolution of the identity for hydrochloric acid in water (left) and benzene (right). Results are shown for OQS-PCM(ω), BO-sol, and the cutoff approaches. The x axis is sampled in increments of 50 states and includes the point corresponding to the full space of singly excited singlet states (172 states for HCl with the 6-31+G(d,p) basis set). On the plotted scale, the cutoff (dark green) and fitted cutoff (magenta) results for benzene are superimposed.

S3.5 Comparison of Solute-Solvent Dispersion with SMSSP Approach

Here, we compare the solute-solvent dispersion contribution obtained with OQS-PCM(ω) with that predicted by an established model in the literature, namely the "Solvation Model with State-Specific Polarizability" (SMSSP) of Marenich, Cramer and Truhlar.[21] Within this model, the dispersion correction to the excitation energy is expressed as:

$$\Delta E_{0I,\mathrm{disp}} = -\sigma \frac{\epsilon_d - 1}{\epsilon_d + 2} (\alpha_I - \alpha_0) \ , \tag{S56}$$

where α_I and α_0 are the gas-phase polarizabilities of the excited state I and the ground state, respectively. The factor $(\epsilon_d - 1)/(\epsilon_d + 2)$ is proportional to the Lorentz-Lorenz function of a spherical dielectric object representing a solvent molecule. Here, the solvent is assumed to be non-magnetic and non-dissipative. Finally, σ is an empirical parameter obtained by fitting this expression to experimental dispersion energies.

To evaluate the SMSSP dispersion contribution, gas-phase polarizabilities are computed using Gaussian 16 at the PBE0/6-31+G(d,p) level of theory, taking advantage of the availability of analytical polarizabilities for both ground and excited states, which are not currently implemented in GAMESS-US. Because the LC-ξBPBE functional is not available in Gaussian 16, the exchange–correlation functional is changed to PBE0 for these calculations. For consistency, OQS-PCM(ω) results are also recomputed at the PBE0 level.

The OQS-PCM(ω) and SMSSP results for the first excitation of the studied systems are reported in Table S2. Dispersion contributions within OQS-PCM(ω) are found to be robust with respect to the choice of functional, with average differences of only ~ 0.002 eV across the systems considered.

A good qualitative agreement between the SMSSP model and OQS-PCM(ω) is observed for HCl (in both water and benzene), as well as for CH_2O and HONO in benzene. In contrast, a pronounced discrepancy is found for CH_2O and HONO in water. In the case of CH_2O in

water, the disagreement also affects the sign of the dispersion contribution. For HONO and CH_2O, OQS-PCM(ω) predicts a change in both the magnitude and the sign of the dispersion correction upon changing the solvent from water to benzene that is not captured by the SMSSP model. For HCl, although both approaches yield the same qualitative trend when changing solvent (i.e., a larger dispersion correction in the weakly polar solvent), a significant quantitative difference remains between OQS-PCM(ω) and SMSSP estimates.

The origin of these discrepancies is twofold. First, the SMSSP model does not account for the *frequency-resolved response* of the solvent to solute fluctuations. Second, it relies on a *dipolar interaction model* between solute and solvent molecules, each of which is considered *spherically symmetric*.

Table S2: Comparison of solute-solvent dispersion energy contribution to the first excitation energy (eV) of molecules in solution according to OQS-PCM(ω) and SMSSP.[21]

System	SMSSP – PBE0	OQS-PCM(ω) – PBE0	OQS-PCM(ω) – LC-ξBPBE
HCl in water	-0.13 eV	-0.24 eV	-0.25 eV
HCl in benzene	-0.17 eV	-0.37 eV	-0.38 eV
CH_2O in water	-0.01 eV	0.15 eV	0.15 eV
CH_2O in benzene	-0.01 eV	-0.03 eV	-0.03 eV
HONO in water	0.00 eV	0.13 eV	0.14 eV
HONO in benzene	0.00 eV	-0.01 eV	0.01 eV

References

(1) Gaspard, P.; Nagaoka, M. Non-Markovian stochastic Schrödinger equation. *J. Chem. Phys.* **1999**, *111*, 5676.

(2) Guido, C.; Rosa, M.; Cammi, R.; Corni, S. An open quantum system theory for polarizable continuum models. *J. Chem. Phys.* **2020**, *152*, 174114.

(3) Ke, Y.; Zhao, Y. Hierarchy of forward-backward stochastic Schrödinger equation. *The Journal of Chemical Physics* **2016**, *145*, 024101.

(4) Tomasi, J.; Mennucci, B.; Cammi, R. Quantum mechanical continuum solvation models. *Chem. Rev.* **2005**, *105*, 2999–3094.

(5) Corni, S.; Cammi, R.; Mennucci, B.; Tomasi, J. Electronic excitation energies of molecules in solution within continuum solvation models: Investigating the discrepancy between state-specific and linear-response methods. *J. Chem. Phys.* **2005**, *123*, 134512.

(6) Onsager, L. Electric moments of molecules in liquids. *J. Am. Chem. Soc.* **1936**, *58*, 1486– 1493.

(7) Barca, G. M. J. et al. Recent developments in the general atomic and molecular electronic structure system. *J. Chem. Phys.* **2020**, *152*, 154102.

(8) Biancorosso, L.; D'Antoni, P.; Corni, S.; Stener, M.; Coccia, E. Time-dependent quantum/continuum modeling of plasmon-enhanced electronic circular dichroism. *The Journal of Chemical Physics* **2024**, *161*, 214104.

(9) Fiedler, J.; Boström, M.; Persson, C.; Brevik, I.; Corkery, R.; Buhmann, S.; Parsons, D. Full-Spectrum High-Resolution Modeling of the Dielectric Function of Water. *J. Phys. Chem. B* **2020**, *124*, 31033113.

(10) Hayashi, H.; Hiraoka, N. Accurate Measurements of Dielectric and Optical Functions of Liquid Water and Liquid Benzene in the VUV Region (1-100 eV) Using Small-Angle Inelastic X-ray Scattering. *J. Phys. Chem. B* **2015**, *119*, 56095623.

(11) Gil, G.; Corni, S.; Guido, C. A. OQSolv: A scientific Python package implementing the Open Quantum System Polarizable Continuum Model in its full frequency-dependent formulation (OQS-PCM(ω)). `https://github.com/LIMElab-theochem/OQSolv`, 2024; GitHub repository. Accessed: 2026-07-16.

(12) Inc., W. R. Mathematica, Version 11. `https://www.wolfram.com/mathematica`, Champaign, IL, 2024.

(13) Avila Ferrer, F. J.; Santoro, F. Comparison of vertical and adiabatic harmonic approaches for the calculation of the vibrational structure of electronic spectra. *Phys. Chem. Chem. Phys.* **2012**, *14*, 13549–13563.

(14) Cerezo, J.; Santoro, F. FCclasses3: Vibrationally-resolved spectra simulated at the edge of the harmonic approximation. *Journal of Computational Chemistry* **2023**, *44*, 626–643.

(15) Santoro, F.; Cerezo, J. FCclasses3, a code for vibronic calculations. 2022; [Online; accessed 14-November-2025].

(16) Cammi, R.; Corni, S.; Mennucci, B.; Tomasi, J. Electronic excitation energies of molecules in solution: State specific and linear response methods for nonequilibrium continuum solvation models. *J. Chem. Phys.* **2005**, *122*, 104513.

(17) Guido, C. A.; Chrayteh, A.; Scalmani, G.; Mennucci, B.; Jacquemin, D. Simple Protocol for Capturing Both Linear-Response and State-Specific Effects in Excited-State Calculations with Continuum Solvation Models. *Journal of Chemical Theory and Computation* **2021**, *17*, 5155–5164, PMID: 34224244.

(18) Gil, G.; Pipolo, S.; Delgado, A.; Rozzi, C. A.; Corni, S. Nonequilibrium Solvent Polarization Effects in Real-Time Electronic Dynamics of Solute Molecules Subject to Time-Dependent Electric Fields: A New Feature of the Polarizable Continuum Model. *Journal of Chemical Theory and Computation* **2019**, *15*, 2306–2319, PMID: 30860829.

(19) Caricato, M.; Mennucci, B.; Tomasi, J.; Ingrosso, F.; Cammi, R.; Corni, S.; Scalmani, G. Formation and relaxation of excited states in solution: A new time-dependent polarizable continuum model based on time-dependent density functional theory. *J. Chem. Phys.* **2006**, *124*, 124520.

(20) Cossi, M.; Barone, V. Time-dependent density functional theory for molecules in liquid solutions. *The Journal of Chemical Physics* **2001**, *115*, 4708–4717.

(21) Marenich, A. V.; Cramer, C. J.; ; Truhlar, D. G. Uniform Treatment of Solute–Solvent Dispersion in the Ground and Excited Electronic States of the Solute Based on a Solvation Model with State-Specific Polarizability. *J. Chem. Theory Comput.* **2013**, *9*, 3649–3659.
